# Constrained neuro fuzzy inference methodology for explainable personalised modelling with applications on gene expression data

**DOI:** 10.1038/s41598-022-27132-8

**Published:** 2023-01-09

**Authors:** Balkaran Singh, Maryam Doborjeh, Zohreh Doborjeh, Sugam Budhraja, Samuel Tan, Alexander Sumich, Wilson Goh, Jimmy Lee, Edmund Lai, Nikola Kasabov

**Affiliations:** 1 Knowledge Engineering and Discovery Research Innovation (KEDRI), School of Engineering Computer and Mathematical Sciences, Auckland University of Technology, Auckland, New Zealand; 2 School of Population Health, The University of Auckland, Auckland, New Zealand; 3 School of Psychology, The University of Waikato, Hamilton, New Zealand; 4 Lee Kong Chian School of Medicine, Nanyang Technological University (NTU), Singapore, Singapore; 5 Department of Psychology, Nottingham Trent University, Nottingham, UK; 6 Center for Biomedical Informatics, Nanyang Technological University (NTU), Singapore, Singapore; 7 School of Biological Sciences, Nanyang Technological University (NTU), Singapore, Singapore; 8 Institute for Mental Health, Singapore, Singapore; 9 Intelligent Systems Research Center, Ulster University, Derry, UK; 10 Institute for Information and Communication Technologies, Bulgarian Academy of Sciences, Sofia, Bulgaria

**Keywords:** Data mining, Machine learning

## Abstract

Interpretable machine learning models for gene expression datasets are important for understanding the decision-making process of a classifier and gaining insights on the underlying molecular processes of genetic conditions. Interpretable models can potentially support early diagnosis before full disease manifestation. This is particularly important yet, challenging for mental health. We hypothesise this is due to extreme heterogeneity issues which may be overcome and explained by personalised modelling techniques. Thus far, most machine learning methods applied to gene expression datasets, including deep neural networks, lack personalised interpretability. This paper proposes a new methodology named personalised constrained neuro fuzzy inference (PCNFI) for learning personalised rules from high dimensional datasets which are structurally and semantically interpretable. Case studies on two mental health related datasets (schizophrenia and bipolar disorders) have shown that the relatively short and simple personalised fuzzy rules provided enhanced interpretability as well as better classification performance compared to other commonly used machine learning methods. Performance test on a cancer dataset also showed that PCNFI matches previous benchmarks. Insights from our approach also indicated the importance of two genes (ATRX and TSPAN2) as possible biomarkers for early differentiation of ultra-high risk, bipolar and healthy individuals. These genes are linked to cognitive ability and impulsive behaviour. Our findings suggest a significant starting point for further research into the biological role of cognitive and impulsivity-related differences. With potential applications across bio-medical research, the proposed PCNFI method is promising for diagnosis, prognosis, and the design of personalised treatment plans for better outcomes in the future.

## Introduction

In molecular genetics, high-throughput gene expression profiling methods, such as microarray and RNA-sequencing are often used to examine the transcriptomic changes between case (patient) and control groups in comparative studies of complex diseases. It is also widely understood that complex, polygenic diseases often do not have clear signals characterised by only a few genes^1^. Over the past decade, there has been numerous attempts in combining machine learning methods with high-throughput gene expression data for diagnosis with promising results such as in the detection of cancer^2^, mental illnesses^3^, and genetic disorders^4,5^. A further objective would be predictive medicine where attempts are made to predict disease progression and response to intervention^6^.

Appropriate modelling of gene expression data can help detect differential gene activations, leading to the identification of critical biological pathways even before the disease is fully developed (at the prodrome phase) which can be used for better informed interventions. However, high-throughput gene expression datasets are high dimensional and usually have a comparatively small sample size^7^. Challenges ensue, as some machine learning models tend to overfit, and the ‘black-box’ nature of some models make it difficult to reveal the underlying biological mechanisms represented in the data. The inherent heterogeneity of some genetic conditions represented in the data can also exacerbate the problem of confounding and lack of transparency, resulting in the model making correct decisions via incorrect reasoning. To trust the results of a machine learning model, interpretable methods are needed, in which the models explain why certain diagnostic/prognostic outputs are produced^8^.

Previous research has shown that fuzzy inference systems are a convenient class of methods that can explain the decision-making process of the model in a systematic manner^9,10^. Neuro-fuzzy Inference systems (NFIS) facilitate automated rule learning from data, which can generate rules characterised by descriptive language involving fuzzy predicates. Knowledge in a NFIS is expressed as IF–THEN rules, with forward chaining propositions involving fuzzy sets. The presence of ‘fuzziness’ allows the NFIS to capture the ambiguity of descriptive language for closer resemblance of human decision making. Therefore, the IF–THEN rule based reasoning, facilitates linguistic representations of the decision-making process of the classifier via natural language. However, fuzzy rules acquired from a NFIS, usually require further adaptation to obtain concise and interpretable rules that can be used in clinical settings to advance biological knowledge^11^.

Under the assumption that the learned rules are applicable to all new data samples, most NFIS approaches aim to learn global rules using the entire training set. However, due to the heterogenous nature of some conditions (e.g., cancer, schizophrenia), global rules may not be appropriate for all genetic subgroups. In some NFIS, local cluster-based IF–THEN rules have been extracted, for example in relation to gene expression patterns^12,13^. Another approach is to build “personalised” machine learning models for each individual using data from other related individuals, selected with respect to clustering criteria^12^. This can generate individual profiles that further enhance the interpretability and allow the development of precision medicine and treatments tailored for every individual. Methodologies for integrating personalised modelling with NFIS have been developed, such as TWNFI and TWRBF^14,15^. The TWNFI and TWRBF models perform well in terms of prediction accuracy, however, the extracted fuzzy rules are not optimized in terms of providing interpretability.

**Figure 1:**
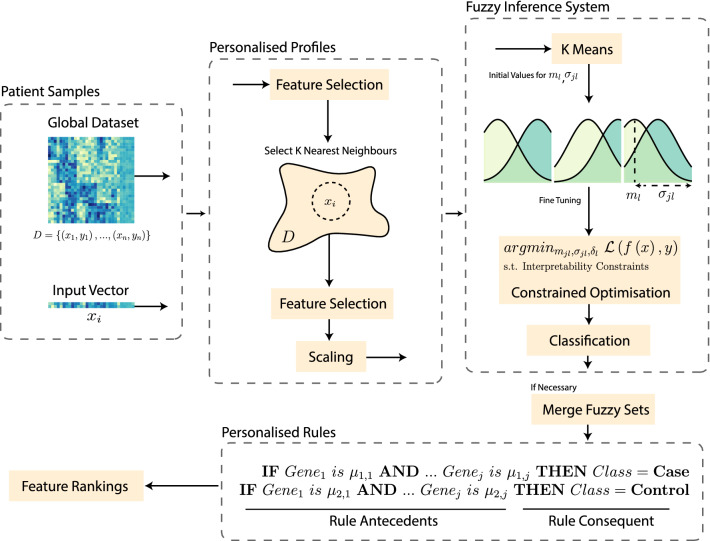
The proposed personalized constrained neuro fuzzy inference (PCNFI) methodology. First, a personal profile is built for the input patient by selecting K Nearest Neighbours (KNN) from the training set. The first layer of feature selection helps in selecting the best neighbours and the second layer (after KNN) helps to improve the structural interpretability of the rules. Next, the fuzzy inference system is initialised by K-Means and fine-tuned via constrained optimisation to ensure semantic interpretability. Following this, redundant fuzzy sets can be merged if the number of rules is high. Finally, personalised fuzzy rules can be extracted based on the genetic profiles of individuals.

Considering these limitations, this paper proposes a new methodology outlined in Fig. 1 to address the interpretability related challenges of personalised neuro fuzzy models on high dimensional gene expression datasets. The proposed new method is a personalised constrained neuro fuzzy inference (PCNFI) model for extracting personalised rules, tailored, and optimised for individuals. The resulting rules are comprehensible, short and concise, fulfilling the conditions of semantic and structural interpretability.

### Main

In fuzzy inference systems, interpretability refers to the readability and comprehensibility of the extracted rules. The general interpretability criterion is defined by two facets, the structural aspects, and semantic aspects of fuzzy rules. For a thorough treatise regarding interpretability conditions of FIS, we refer the reader to^16–18^. The methodology proposed in this paper focuses on the implementation of these conditions and extracting rules based on personalised profiles of the patients.

### Structural interpretability

The structural interpretability aspect focuses on the readability of the rules, this mainly refers to the components that describe the model complexity, including number of rules, number of rule antecedents (features), and number of membership functions. Fuzzy rules in a conventional system include all features in its premise as antecedents. Of course, the rules will be easier to understand if they are defined by only the most important features in the data. A common method is to perform feature selection (e.g., correlation, entropy) before defining the fuzzy partitions. Alternatively, some studies considered a more localised ‘rule-by-rule' approach by having a ‘don’t-care’ clause, indicating a uniform membership over all elements in the universal set^19,20^. However, this is not applicable for NFIS with gradient based optimisation. Rule-base simplification or reducing the number of rules is also essential for complexity reduction. The simplest approaches utilise incremental procedures which start with a small rule base and incrementally add more rules until the model accuracy converges or a threshold is reached. Conversely, it is also possible to start with a high number of rules and incrementally shorten the rule base^21^. Genetic or evolutionary approaches have also been utilised to tackle multiple, conflicting objectives to find the optimal trade-offs between minimising the number of rules and maximising the accuracy^22^. Other methods which may include merging similar rules using similarity metrics, possibility measures or disregarding redundant rules have also been applied^23^. Structural interpretability improves the rule readability and are commonly applied to NFIS models. However, semantic interpretability is often neglected.

### Semantic interpretability

For a fuzzy model to be interpretable, it is required that the semantics of the learned rules and those known naturally to the users are coherent. Simply put, reading the linguistic/symbolic representation of the rules should make logical sense and relate to some meaning^17^. Common conditions for semantic interpretability include distinguishability, coverage, and relational preservation^18^ (see Supplementary Fig. S1). To satisfy these requirements, the fuzzy sets can be defined as fixed, such that semantic interpretability criteria is naturally fulfilled. For fine-tuning of fuzzy sets during training, constraints are typically imposed on the objective function to ensure that the semantic interpretability criterion is met*.* For constrained optimization, a penalty term is introduced to the objective function to drive similar fuzzy subsets together and eventually merge them. This brings in twofold benefits by improving the distinguishability and lowering the number of membership functions^24,25^. Constraints can be enforced to ensure non-negative widths for the gaussian membership functions and to bound the gaussian centres within the universe of discourse (UOD). Some of these conditions require the use of inequality constraints, which can make optimisation difficult due to the non-convex and non-linear nature of NFIS loss functions. Previous studies attempt to clip these variables via IF-ELSE conditions^26^. However, this may result in unstable training and non-optimal solutions. To overcome these issues, the PCNFI method utilises the log barrier method used in inequality constrained optimisation problems. The log barrier belongs to family of interior-point methods, which approximate Lagrangian optimization as a sequence of unconstrained problems^27^. This helps facilitate easy training with standard gradient-based methods.

### Personalised predictive modelling

Personalised modelling is based on transduction, to build a model on data samples that are in some way relevant to the input vector^28^. A primary advantage of personalised modelling is that it can identify a cluster of input vectors to build unique profiles for each individual. Developing a system to offer personalised models and profiles for each patient can be quite useful in clinical applications, especially when dealing with high dimensional gene expression datasets that have a heterogeneous nature with molecular subgroups which may exhibit different disease progression across individuals who may require different treatments. Previous predictive personalised models include WWKNN^12^, TWRBF and TWNFI^14,15^. While the previous personalised NFIS based methods showed good performance, the extracted personalised fuzzy rules were not optimised in terms of better interpretability. The rule learning process was not enforced to follow pre-defined semantic and structural interpretability conditions, so that interpretability becomes part of the learning process. In the current paper, the proposed PCNFI method improves the model performance by incorporating interpretability conditions in the methodology.

### Datasets

The PCNFI methodology is applied to two sets of gene expression data for diagnosis of mental illnesses and another Liver cancer gene expression dataset for classifying between tumours and non-tumours tissue.

#### LYRIKS data

This is a cohort study characterizing differences in youth at ultra-high risk (UHR) for psychosis compared to healthy controls. Participants were drawn from mental health care, community-based services, and various educational institutions in Singapore. Healthy controls in this study are participants who did not fulfil the UHR criteria and had no psychiatric history. Gene expression measurements were taken from Peripheral blood; with 34,694 genes (features)^29^. This is a two-class problem comparing healthy controls against UHR. The dataset comprises of 84 total samples with 55 UHR participants and 28 healthy controls. For more information on the acquired gene expression dataset, please refer to^30^.

#### Bipolar data

Bipolar disorder (BD) is a psychiatric disorder characterized by instability in mood, resulting in manic and depressive episodes. This cohort was collected from the Netherlands with 240 controls and 240 cases. Peripheral whole blood was drawn and processed for genotyping and RNA sequencing from 240 controls and 240 cases, of whom 227 and 13 were diagnosed with bipolar disorder type 1 and type 2 respectively. The dataset has 20,583 genes (features) after pre-processing. This is also a two-class classification problem, controls vs Bipolar cases with a total of 467 samples. For more information on the acquired gene expression dataset, please refer to^31^.

#### Liver cancer data (GSE57957)

This dataset is based on the expression profiling of tumor and adjacent non-tumorous tissues of Hepatocellular Carcinoma (HCC) patients, a common type of liver cancer. The National Cancer Centre of Singapore (NCCS)/SingHealth Tissue Repository provided tissues of the HCC patients. Samples included 59 tumours and 59 adjacent non-tumorous samples. The dataset has 47,325 genes (included non-annotated genes). For more information, please refer to^32^ This is also a two-class classification task with the aim to classify between tumours and adjacent non-tumorous tissues of HCC patients. Although, this is not a diagnosis problem, we have used this example to show that the classification performance of PCNFI can be generalized to other (non-mental health) diseases.

## Results

### Classification performance and interpretability of PCNFI

To test the classification performance and structural interpretability of PCNFI for diagnosis of UHR and bipolar, we try different combinations of the number of features, neighbours and rules. The generalisation of our approach is tested by leave one out cross validation (LOOCV). The unlabelled test vector from each LOOCV split is considered as an input vector (excluded from training), for which a personalised model is built using the labelled training data. For the proposed PCNFI model, the main hyperparameters include, the neighbourhood size (selected from each class), the number of rules and the strength of penalties and log barrier during constrained optimisation. The optimal strengths for the penalties and the log barrier are manually chosen by considering the optimal level of trade-off between the accuracy and interpretability.

**Figure 2:**
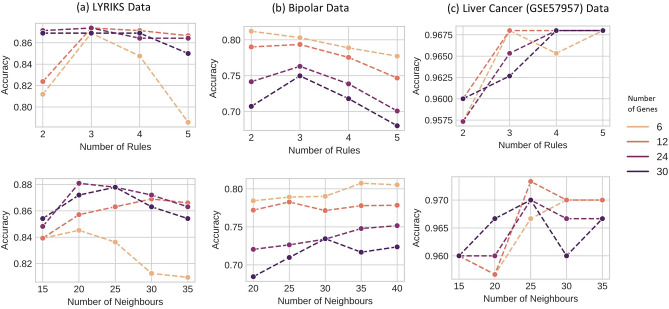
Accuracy given number of rules and neighbours. (**a**) Changes in accuracy for classification of UHR, (**b**) Bipolar subjects and (**c**) HC tissue given different number of rules, neighbours (from each class) and features/genes.

In Fig. 2 for the LYRIKS data (a), increasing the number of rules does not improve the classification accuracy if the number features are high (24, 30). However, with lesser genes, increasing the number of rules to 3 does improve the accuracy. For the bipolar data, a lower number of genes and rules usually achieve the highest accuracy. This shows that for both datasets we are able to obtain fuzzy rules which are short and consice. Another interesting observation is that for BP dataset which contains 467 samples, we can obtain good accuracy by only using a fraction of samples to train the model.

The PCNFI model is also compared to other popular classification techniques including Support Vector Machine (SVM), Naïve Bayes, Gradient Boosted Trees and Multi-layered perceptron. Feature selection is performed separately in each LOOCV split by ranking the features using signal to noise ratio method (SNR)^33^ on the training dataset and using the same features on the unlabelled test set. The Hyperparameters are tuned via a Bayesian optimisation approach, called tree-structured parzen estimator^34^. This method approaches hyperparameter optimisation problem from a probability perspective and uses past trails to choose the next best set of hyperparameters for evaluation.

**Figure 3:**
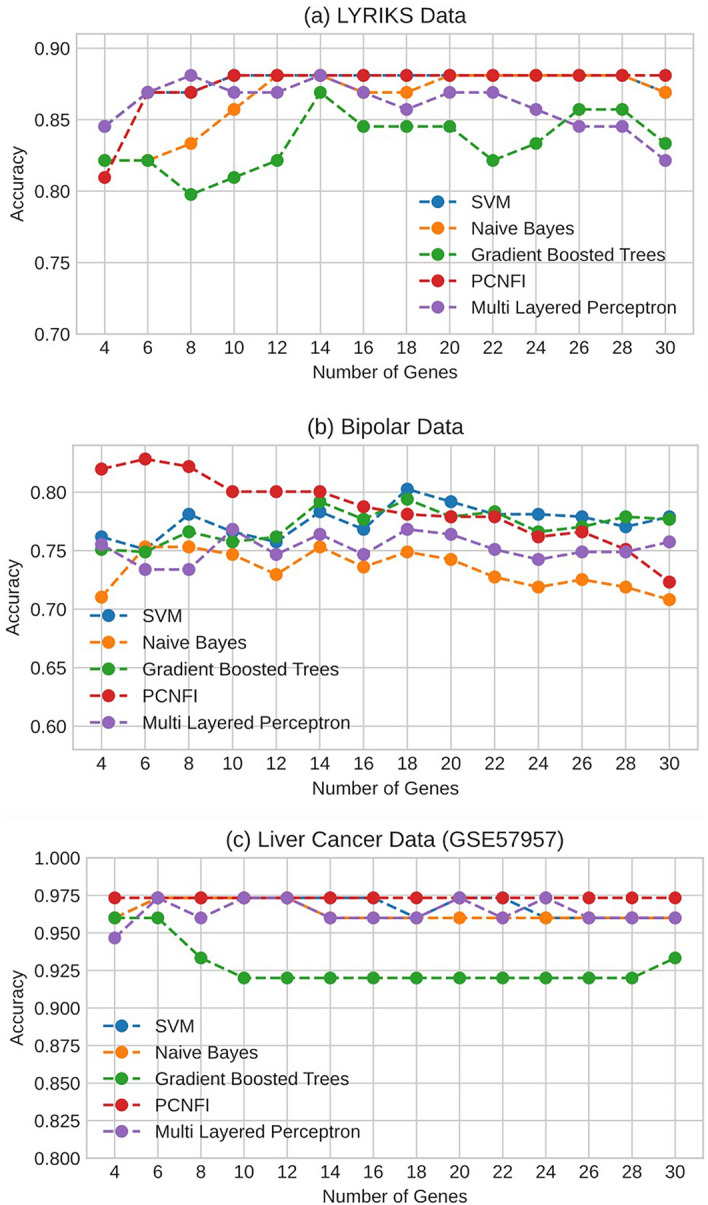
Classification accuracy of PCNFI compared with other methods. (**a**) Classification accuracy on the LYRIKS dataset into UHR vs control groups (**b**) the Bipolar dataset into bipolar versus control groups (**c**) and Liver cancer dataset with an increasing number of features (genes) selected by SNR for every personalised model. Features can be different for each model, but many are presumed to be similar across individual models.

Figure 3 shows the classification accuracy of the PCNFI model in comparison with other machine learning approaches. The accuracy grows by increasing the number of top selected genes and it converges to the highest point and sustains after 10 genes. In Fig. 3a, the LYRIKS dataset required 3 rules and 10 antecedents to achieve the highest accuracy, while the bipolar dataset Fig. 3a required 2 rules and 6 antecedents to achieve the highest accuracy. For the bipolar dataset Fig. 3b, top 6 features ranked by SNR on a neighbourhood of 35 samples per class (selected from Fig. 2b) gave the highest accuracy, meaning 6 antecedents per rule. As compared to other models in Fig. 3, PCNFI requires only a small number of rules and rule antecedents (10 or less) to achieve the same or better classification accuracy. This shows that rules from PCNFI which are structurally interpretable, can be used to classify UHR and Bipolar subjects. Results in Fig. 3c showed that gene expression from tumours tissues can be easily differentiated from adjacent non-tumours tissues. In this example, PCNFI consistently performs well across all different number of genes and meets the previous benchmark reported in^35^. Comparisons using other metrics (F1 Score, Precision, Recall) are reported in Supplementary Fig. S4 and Supplementary Tables S1–S3.

### Improved semantic interpretability of the rules

**Figure 4:**
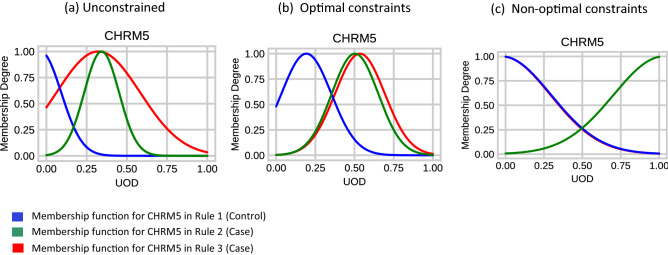
Comparing the effect of constraints on the interpretability of a feature/gene. The three plots (**a**), (**b**) and (**c**) show the three membership functions (belonging to three rules) for the CHRM5 gene, when no constraints are active, optimal constraints are active and non-optimal constraints are active.

The previous section has shown that PCNFI can achieve good accuracy with structural interpretability by having a small number of rules and rule antecedents. Next, we demonstrate the semantic interpretability improvements given by PNCFI through three examples in Fig. 4. The semantic constraints are applied on the model parameters which are fine-tuned during training. To illustrate the effectiveness of these constraints, we first take an example gene (CHRM5) and its membership functions from the set of three rules extracted for a sample from the LYRIKS dataset. Then, we compare how the membership functions for this feature change when the model is trained with no constraints as in regular NFIS (Fig. 4a) and when constraints are applied in PCNFI (Fig. 4b,c). In Fig. 4a with no constraints, the fine-tuned membership function in green is completely contained within another set and the centre of a membership function (in blue) is outside the range of 0 and 1. In this case, the rules lack comprehensibility as genes, when scaled between 0 and 1, cannot be outside this range. Moreover, the membership functions are not logically coherent and cannot be represented linguistically due to being contained in one another.

Figure 4b shows the same variable and its membership functions when the semantic constraints are activated. The constraints are forcing the membership functions to have similar widths and distinct centres, this has resulted in an improvement in relational preservation and distinguishability between the membership functions. Therefore, the rules now have a logically coherent linguistic representation. Moreover, the membership functions in red and green can now be merged to reduce redundancies as they have become very similar to each other. As shown in Table 1, we also observe an increase in the accuracy. The strengths of the constraints can be tuned to find the desired trade-off between interpretability and accuracy. As an example, we show that when the strength of the constraints is high as in Fig. 4c, the centres of the membership functions are forced to be as distinct as possible within the bounds of the universe. Although, while there is an improvement in the distinguishability there is a loss in accuracy and the membership functions are too ambiguous losing their meaning. This shows the trade-off between accuracy and interpretability that can be achieved by tuning constraint strengths as hyperparameters in PCNFI. The updates to membership functions during training are visualised in Supplementary Fig. S2.

**Table 1:** Comparing accuracy and constraint strength.

Constraint status	No constraints	Optimal constraints	Non-optimal constraints
Penalty strengths	α1 = 0, α2 = 0	α1 = 0.1, α2 = 0.01	α1 = 0.6, α2 = 0.08
Accuracy	0.8095	0.8214	0.7976

The effect of *different penalty strengths* on the classification accuracy of the *LYRIKS dataset* (accuracy using top 8 *genes*).

### Symbolic representation of the rules

One of the main advantages of fuzzy inference systems is the possibility for symbolic representation of the rules. The symbolic representation can help describe the general behaviour of the system in natural language. The fuzzy sets utilised for rule learning in this paper are not fixed and are fine-tuned by minimising an error. This makes linguistic labels such as ‘high’, ‘low’ unsuitable as the support (range) for these variables is not properly defined. Instead, we describe the system in other imprecise terms such as ‘about’ or ‘approximately’. A fuzzy set in an antecedent condition is described as ‘gene 1’ is about ${m}_{jl}$, this represents a membership that is symmetric around the centre of the membership function with some ambiguity represented by the gaussian width ${\sigma }_{jl}$. Input values which are closer to the centre will contribute more towards the firing strength of a rule. As each rule should represent the different fuzzy regions, we look at the firing strength and the δ parameter to determine which class is associated with each rule. We demonstrate an example of the symbolical representation of rules with 8 antecedents from the LYRIKS and 6 antecedents from the Bipolar datasets. Tables 2, 3 show examples of the symbolic representation of rules from a randomly selected sample from the LYRIKS and Bipolar data respectively.

**Table 2:** Example rule from the LYRIKS dataset.

	LOC730535	HS.143909	ATRX	HS.580154	CHRM5	HS.553290	CCDC49	HS.377021	Class
Rule 1	0.5301	0.7419	0.4915	0.7823	0.2240	0.6872	0.2326	0.6839	Case (UHR)
Rule 2	0.7517	0.4769	0.7145	0.5512	0.1717	0.4141	0.5885	0.5	Control
Rule 3	0.3289	0.7419	0.3385	0.7823	0.4288	0.6872	0.2705	0.8029	Case (UHR)

Example of personalised classification rules (in symbolic form) from a randomly selected sample in the LYRIKS dataset. The columns represent the antecedents of each rule. Antecedents are expressed by ‘about’ terms, e.g., Rule 1: IF (LOC730535 is about 0.5301) AND … AND (HS.377021 is about 0.6839) THEN Class is Case, etc.

**Table 3:** Example rules from the BP dataset.

	TSPAN2	MIR23AHG	FAR2	MAK	RANBP2	TSR1	Class
Rule 1	0.1272	0.1809	0.2157	0.2198	0.5859	0.5760	Control
Rule 2	0.7163	0.6611	0.7300	0.5832	0.3432	0.3619	Case (Bipolar)

Personalised classification rules from a randomly selected sample in the BP dataset.

Table 2 shows that the ATRX gene is up-regulated for the control class and down-regulated for the case class. This is very interesting as literature has also shown that reduced ATRX is associated with loss of H3K9me3 and telomere lengthening—a hallmark of many cancers. It is likely that inactivation of ATRX in postmitotic neurons, following neurogenesis and lamination, will help define a role for ATRX target genes in altered synaptic activity and/or synaptic plasticity underlying cognitive impairment^36^. MRI studies on ATR-X patients (syndrome caused by mutations on the ATRX gene) showed severe glial defects and white matter disruption^37^.

### Visualisation of personalised rule profiles

**Figure 5:**
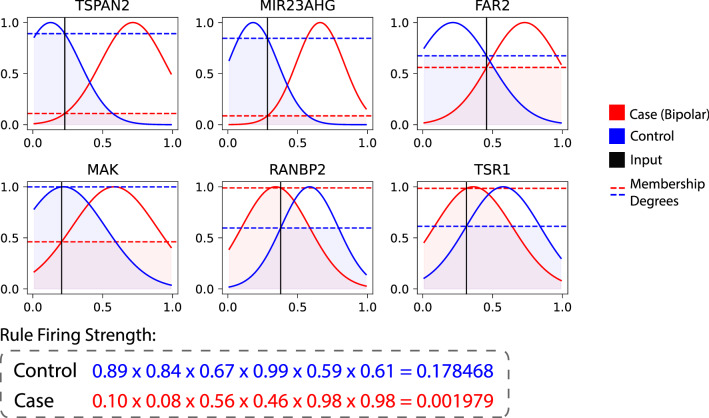
Visualisation of personalised rule profile from a random selected sample (individual) in the bipolar data. Each plot shows the genes and the associated membership functions. The colours red and blue distinguish between the two rules representing case and control respectively. The black vertical lines represent the observed gene expression values of the individual. The horizontal lines represent the degrees of membership of each input with respect to the antecedents and the associated fuzzy sets. The individual is correctly classified by the proposed PCNFI in the class of Control (in blue).

For another perspective, the personalised rule profiles can be visualised to show how rules are activated for an individual (a randomly selected sample from the bipolar data). The example in Fig. 5, shows the rule profile for an individual derived from neighbouring samples with known outcomes. We can observe that higher gene expression values for all genes (except for RANBP2 and TSR1) indicates a case class (which is bipolar in this dataset). Given the gene expression values of the individual (Fig. 5, black vertical lines) the degrees of memberships for the rule in red (representing case class) are very low for the TSPAN2, MIR23AHG genes*.* Contrarily, the degree of memberships for rule representing the control class are high. For the RANBP2 and TSR1 genes the inputs have higher memberships in the sets representing the case class. However, since other sets representing the control class have membership close to zero, the effect of these genes (RANBP2 and TSR1) on the overall outcome gets mitigated. As the propositions are linked with a product operator the resulting firing strength for this rule is very low and the individual is correctly classified as control.

### Frequently selected genes

The personalised model for each input sample is created by selecting genes with respect to the nearest neighbouring samples to the input. This may result in unique sets of genes being selected for each sample. It is expected that there will be some overlap between selected gene sets across different personalised models, signifying possible common origins or functional overlaps. Indeed, deepened insights into those genes which are frequently selected amongst the top sets of discriminatory genes may provide information regarding their potential to serve as diagnostic biomarkers for mental health status progression. The size of the neighbourhoods for the LYRIKS data was 30 samples from each class and for the bipolar dataset, 35 samples from each class. We created a total of 84 personalised models corresponding to 84 samples in LYRIKS dataset and 467 Personalised models corresponding to 467 samples in bipolar dataset. Table 4 shows top 10 genes with the highest frequency of being in the gene sets selected from each of these personalised model’s neighbourhood. The gene sets selected from each neighbourhood includes top 10 genes for the LYRIKS dataset and top 6 genes for the bipolar dataset ranked with highest SNR, which give the highest possible accuracy (both in the UHR group of LYRIKS data and the bipolar data). As shown in Table 4, among the 84 personalised models built for the LYRIKS dataset, the ATRX gene appeared among the top 10 selected gene sets in 79 models. For the Bipolar dataset, TSPAN2 was ranked within the top 6 genes for 433 neighbourhoods out of a total of 467. This indicates the importance of the ATRX and TSPAN2 genes as possible biomarkers for differentiating the UHR, bipolar and healthy individuals^38^.

**Table 4:** The frequency of genes being selected among the top 10 genes for LYRIKS and top 6 genes for BIPOLAR (selected by SNR) for every individual neighbourhood in the LYRIKS and Bipolar dataset.

LYRIKS dataset		BP dataset	
Gene	Frequency	Gene	Frequency
ATRX	79	TSPAN2	415
CTDSPL2	72	FAR2	337
HS.143909	68	CNTNAP3	188
ARID4B	68	MIR23AHG	173
HS.377021	55	LINC01765	166
LOC730535	51	SLC25A20	165
CHRM5	46	MAK	165
LOC644162	43	SERPINF1	150
CCDC49	38	LILRA4	145
LOC401623	38	CPT1A	120

We created a total number of 84 personalised models for the 84 samples in LYRIKS datasets and 476 Personalised models for the 476 samples in bipolar dataset.

## Discussion

The reasoning behind the output of a classification system (diagnosis) is essential for clinical and biological application of gene expression datasets. Henceforth, this paper has proposed a new method called PCNFI, to better understand the decision-making process for gene expression classification via interpretable personalized rules. The contribution of this study is twofold, first is the novel PCNFI methodology itself and second is the biological findings obtained using this method on two gene expression datasets (LYRIKS and bipolar). The main component of the PCNFI method is the implementation of semantic constraints using constrained optimisation. Some semantic conditions like the boundedness of UOD require the use of inequality constraints. In general, optimising with such constraints is difficult due to the non-convex and non-linear nature of NFIS losses. A common work around used in previous studies is to clip the variable if it violates the UOD range. However, this may result in unstable training and non-optimal solutions. In this work we have implemented the inequality constraints using the log barrier method which approximates constrained optimization as an unconstrained problem. This leads to all constraints being fully handled by gradient descent optimisation as in standard unconstrained losses. Overall, PCNFI implements two other constraints as penalties to maintain distinguishability and relational preservation between fuzzy sets. Consequently, the resulting rules have been shown to be more interpretable while maintaining good accuracy. Another essential component of our methodology is personalised modelling, which means that rules are personalised for every individual based on their genetic profile. Based on the profiles, the rule antecedents vary for individuals in terms of which genes are included and the conditions which causes the rule to fire.

The biological findings of this paper include the ATRX and TSPAN2 genes as possible biomarkers for differentiating the UHR, bipolar and healthy individuals^38^. The ATRX gene is involved in transcriptional regulation via the chromatin remodelling process^39^. According to prior research, the ATRX gene appears to regulate the activity of two genes: HBA1 and HBA2, which are required for the production of haemoglobin protein, which transports oxygen throughout the body^40^. Therefore, ATRX gene mutations are associated to X-linked syndromes with cognitive disabilities as well as alpha-thalassemia (ATRX) syndrome. Impaired oxygen transport may result in chronic low-level ischemia, to which, regions implicated in a mechanistic cascade to psychosis e.g., hippocampus^41–43^, are particularly vulnerable^44^. Reduced expression of ATRX is associated with hippocampal dysfunction^45,46^ and a neurodevelopmental syndrome^47^. However, to our knowledge we are the first to associate ATRX with risk for psychosis.

Tetraspanin 2 (Tspan2) is typically localised to compact myelin. Its gene expression and exon usage are increased in the mesocorticolimbic pathway (nucleus accumbens) of highly impulsive Wistar rats^48^. This may reflect involvement in oligodendrocyte development and neuroinflammation^49^, which is a risk factor for impulsivity, emotional dysregulation and psychopathology^50^. Impulsivity is high in several psychological conditions (e.g., bipolar disorder^51^;). Our current findings are novel in highlighting the importance of Tspan2 for Bipolar disorder in humans and represents a critical starting point for further research. For example, together with mania, hyperlocomotion and excitement, impulsivity contributes to an ‘excitement’ symptom cluster associated with specific neurophysiological functions^52^, also seen in the very early stages of psychosis^53^, that should be investigated in relation to Tspan2 in future studies.

It should be noted that there are limitations to our approach. The PCNFI methodology has utilised SNR to select the most important genes. While the advantage of SNR is that it is fast, it may not be the best way to select top genes. SNR is a univariate feature selection method which does not account for the interactions between the genes. For future work, other gene selection methods like DeSEQ2^54^ and RFE-SVM^55^ could also be tested and compared against SNR in terms of stability and reproducibility however, such benchmarks would require proper gold-standard evaluation data, which falls outside the scope of this work. Another limitation may be our neighbourhood selection approach which relies on Euclidean distance with uniform weights. Different types of distance functions and varying feature (gene) weights should be explored to define more accurate patient profiles. Gene expression is only one of many biological modalities. Although high-dimensional, within themselves, gene expression alone may not capture fully the richness and completeness of information needed to make confident assertions regarding diagnosis and individualized disease progression. This presents limitations on the quality of the explanations provided by our model. Thus, future work should try to incorporate other OMICS variables (inclusive of proteins and metabolites), other behavioural and clinical variables or demographic variables to help define a more accurate and representative neighbourhood. Another future direction of our research is to extend and apply the proposed PCNFI method to advanced cohort studies that monitor patients’ performance over longitudinal data measurements for early prediction of mental illnesses. This includes gene expression time series data, where the personalised rules in PCNFI will include a time component.

## Conclusion

This paper has presented a new constrained personalised neuro fuzzy inference (PCNFI) methodology for extracting interpretable personalised rules from high-dimensional gene expression datasets. The proposed method allows for structural and semantic interpretability of the fuzzy rules by improving the readability and comprehensibility of the rules. To improve the structural interpretability, this paper has utilised methods for feature selection, clustering and merging of the rules. Semantic interpretability is improved by constrained optimisation during the fine-tuning phase. The effectiveness of the proposed method is demonstrated on two case studies of gene expression datasets. Results indicate that the proposed method not only offers superior interpretability, but also achieves better classification accuracy compared to traditional machine learning algorithms (e.g., SVM, Naïve Bayes, Gradient Boosted Trees). The results showed 82% classification accuracy on the bipolar dataset which shows on-average up to 8% of improvement compared to other classification methods when using only 4 to 8 genes. While showing up to 88% classification accuracy on the LYRIKS dataset when using only 6–20 genes. Performance comparison on the liver cancer gene expression dataset showed that PCNFI matched previously reported benchmarks. In addition to increasing the accuracy, the proposed method extracted personalised rules that are interpretable and can be used to explain certain decisions (diagnosis) given by the model. The rules are also helpful to describe the general behaviour of the system in natural language and through visualisations, hence providing explainability for end-users. Certain genes that regulate mechanisms implicated in risk for psychosis but have never been discovered as associated factors of psychosis, have also been identified in this research. Further research is required to evaluate biological mechanisms through which these genes are associated with risk for psychosis. The proposed method can be potentially used for personalised early diagnosis and prognosis in many other diseases, based on relevant gene expression data.

## Methods

### The proposed PCNFI

Neuro-fuzzy inference systems (NFIS) are types of artificial neural networks based on the principles of fuzzy logic^10^. Hence, they are universal approximators that can perform non-linear mappings between input and output pairs, along with having an “IF X is A, THEN Y is B” rule structure, where A and B are the fuzzy sets associated with the respective antecedents and consequents. Fuzzy logic enables class definitions to be interlaced and can be used for creating meaningful linguistic representations. Unlike classical Boolean logic, which only allows true or false propositions, fuzzy logic allows propositions to have degrees of truth. As a result, the system is more transparent to humans, while also providing additional validation tools for experts.

Neuro fuzzy inference methods are supervised learning methods that enable data-driven rule generation. Given data of the form $D = \left\{ {\left( {{\mathbf{x}}_{1} ,y_{1} } \right), \ldots \left( {{\mathbf{x}}_{n} ,y_{n} } \right)} \right\} \subset {\text{X}} \times {\text{Y}} \}$, where each ${\text{x}}_{j}\in {R}^{d}$ is d-dimensional real-valued vector of attributes (e.g., genes) and ${y}_{j}\in \{\mathrm{1,0}\}$ denotes its corresponding label, the objective is to learn the mapping $f :X \to Y$, the function $f$ is a network with neuro-fuzzy structure described as follows (also illustrated in Supplementary Fig. S3):

1 $$\mu \left( {x_{i} } \right)_{lj} = exp\left( {\frac{{ - \left( {x_{ij} - m_{jl} } \right)^{2} }}{{2\sigma_{jl} }}} \right)$$

2 $$f\left( {x_{i} } \right) = \sum\nolimits_{l = 1}^{M} {\delta_{l} \frac{{\mathop \prod \nolimits_{j = 1}^{d} \mu \left( {x_{i} } \right)_{lj} }}{{\mathop \sum \nolimits_{l = 1}^{m} \mathop \prod \nolimits_{j = 1}^{d} \mu \left( {x_{i} } \right)_{lj} }}}$$

3 $$\hat{y} = \frac{1}{{1 + e^{{ - f\left( {x_{i} } \right)}} }}$$

where $l$ denotes the rule from the total set of rules $M$, $j$ denotes the feature from the set $d$ and $i$ denotes the current sample.

#### Fuzzification layer

Fuzzification determines the degree to which an input belongs to each of the fuzzy sets via membership functions, therefore it is converting crisp values into fuzzy values. In this method, a gaussian membership function (MF) was used, as they are continuously differentiable, this facilitates easy optimisation with gradient based methods. As part of updating the knowledge base (learning procedure) the shape of the gaussian curves can be adapted by tuning parameters ${{\varvec{m}}}_{{\varvec{j}}{\varvec{l}}}, {{\varvec{\sigma}}}_{{\varvec{j}}{\varvec{l}}}$ described in Eq. (1).

#### Rule layer

The rule layer computes the product of the previous layer’s inputs. A node at the rule layer represents the propositions part of a rule that takes d inputs; hence, the product operator allows for modelling interactions between the propositions in the antecedent.

#### Normalisation layer

The normalisation layer computes the firing strength of each rule, reflecting to what extent the input ${\text{x}}_{j}$ satisfies all propositions in the antecedent. When there is a single rule representing every class, the firing strength will indicate which rule was ‘satisfied’ the most.

#### Defuzzification

The defuzzification layer defuzzifies the input from the previous layer into a single crisp value.

#### Output probability

The output from the defuzzification layer is fed into the sigmoid function to output a probability $\widehat{y}$.

The proposed methodology consists of the following steps:

1. Feature selection using SNR to filter out genes with low variance.
2. Selecting nearest neighbours from the data using features selected from step 1.
3. Structural interpretability with a second layer of feature selection and initial fuzzy partitioning.
4. Training a NFI model with constrained optimisation to achieve semantic interpretability.
5. Classification using the trained model and testing the input vector.
6. Merging similar fuzzy sets to reduce redundancy.

Further details of the proposed methodology are explained in the following subsections.

### Initial feature selection and neighbourhood selection

In personalised modelling, a separate model is built on the nearest neighbouring samples to each input vector. Neighbours are selected by considering a distance (usually Euclidean) between the input vector and labelled samples (global dataset). In high dimensional spaces such as gene expression data, distance metrics can become ineffective and may converge to become equidistant. To resolve this problem, we perform a first layer of feature selection to filter out genes with low variance. Then, the distances between an input vector ${x}_{a}$ and other samples ${x}_{b}, \forall b\in D$ in the global data are calculated using Eq. (4). The calculated distances $dist$ are used to obtain K Nearest Neighbours (KNN) that are selected from each class.

4 $$dist = \sqrt {\sum\nolimits_{j = 1}^{d} {\left( {x_{aj} - x_{bj} } \right)^{2} } } , \quad \forall b \in D$$

### Feature selection and initial fuzzy partitioning

According to the model described in Eq. (2), each feature in the dataset is used as an antecedent condition in every rule. This hinders the structural interpretability of the rules as it can result in a huge number of propositions. Short and concise rules are an important condition to take full advantage of the FIS. For reduced number of antecedents, we can discard uninformative genes and consider only discriminant genes, which better distinguish between the two classes and may act as possible biomarkers. This can be achieved via feature selection on the neighbourhood selected in step 2. Among the three major categories of feature selection methods, including filter, embedded, and wrapper methods, a simple filter method is considered here, which selects and ranks features based on the signal-to-noise (SNR) between the classes. Features with maximal difference in mean and minimal variation between the different classes are ranked higher and considered more discriminatory. For a two-class problem the SNR of a feature *j* is calculated as shown in Eq. (5):

5 $$SNR_{j} = \frac{{\left| {mean_{class 1} - mean_{class 2} } \right|}}{{std_{class 1} + std_{class 2} }}$$

Fast computation and lower feature selection bias are the key advantages of a filter approach for feature selection of high-dimensional data. In some cases, wrapper methods can yield better performance as they are multivariate and can account for interactions between features. However, in high-dimensional settings, filter method may give better generalisation as wrapper methods are susceptible to overfitting^56^. Moreover, filter methods are also stable while being accurate^57^. Stability is quite important in the context of personalised modelling as we do not want small perturbations in the data significantly effecting which features are selected. Low stability may result in rules having very different genes for each individual, which is not ideal. For this reason, the proposed methodology in the current paper includes SNR ranking as a more suitable approach since it considers the general properties of the data to rank features.

The other important aspect for structural interpretability is in having a lower number of rules. Each rule in a FIS should ideally reflect a cluster or a fuzzy region where the system’s behaviour is primarily determined by the rule and not by the interpolative behaviour between other rules. For this reason, clustering techniques can be useful for determining the initial fuzzy partitioning. Therefore, in this paper, the K-means clustering algorithm is employed to obtain K cluster centres which are used as initial values for the rules. The initial fuzzy partitioning process is followed by an optimization procedure to fine tune the gaussian membership functions by minimizing the training error. This process is described in the next section.

### Training a constrained NFI model

With a reduced number of rules and rule antecedents, the final system can still lack in semantic interpretability if trained through unconstrained learning. By restricting the tuning of the gaussian membership functions we can ensure that conditions for parameter bounds, distinguishability, coverage, and relational preservation are met (Visualised in Supplementary Fig. S1).

#### Bounded universe of discourse

To maintain the consistency of the membership functions, we ensure that the parameters ${m}_{lj}$ stay within the bounds of the universe of discourse (UOD). The fuzzy system is defined over a multidimensional UOD, that can be decomposed into many one-dimensional UOD, each associated with a feature. With gene expression data it is a common practice to scale the values between 0 and 1, 0 $\le {m}_{lj}\le 1$. Therefore, the centres of the gaussians should stay within this range during and after optimisation.

#### Relational preservation

Also known as proper ordering, this condition is violated if the upper and lower tails of one membership function are fully contained in another membership function. This implies that the semantics of a term are fully contained in another term, resulting in incomprehensibility. In such cases, relations between corresponding fuzzy sets can be preserved by enforcing membership functions to have similar widths. The similarity between different ${\sigma }_{lj}$ can be quantified by considering the distance between the widths as shown in Eq. (6).

6 $$p_{1} = \mathop \sum \limits_{l \ne k \in M} \mathop \sum \limits_{j = 1}^{D} \left( {\sigma_{lj} - \sigma_{kj} } \right)^{2}$$

#### Coverage

This condition requires that each element of the universe of discourse belongs to at least one fuzzy set. Lack of full coverage may result in some input values having no effect on the inference. If an input value does belong to any membership, it will indicate incompleteness, and reduce comprehensibility. To ensure this condition is satisfied, we can initialise the system with higher ${\sigma }_{lj}$; however, this may also result in lower distinguishability.

#### Distinguishability

This condition requires fuzzy sets to be well separated and refer to distinct concepts. If all membership functions assign similar degrees of membership to all elements, the feature will have little or no contribution to the firing strength of the rules. This brings redundancy and semantic confusion as different linguistic labels will refer to same concepts. To quantify the separability or the distinguishability, many approaches have been proposed^58,59^. The most common is the set theoretic based similarity measure (Jaccard similarity), however this measure is computationally expensive due to the integration operation. Therefore, it is not suitable for efficient implementation of gradient-based training. Instead, an analytical measure based on distance (Eq. 7) is more applicable for neuro fuzzy inference systems as it is easily differentiable.

7 $$p_{2} = \mathop \sum \limits_{l \ne k \in M} \mathop \sum \limits_{j = 1}^{D} \frac{1}{{1 + \left( {m_{lj} - m_{kj} } \right)^{2} }}$$

In summary, NFIS with lower ${p}_{1}, {p}_{2}$ will ideally be more semantically interpretable.

#### Constrained optimization

To ensure that the aforementioned conditions for interpretability are met, we can setup the training process as a constrained optimisation problem, to arrive at a personalised constrained NFI (PCNFI). The objective function (loss function) is minimised with respect to some constraints on ${m}_{lj},{\sigma }_{lj}$ to satisfy the conditions. The constraints can either be ‘soft’ by imposing penalties on objective function or ‘hard’ inequality and equality constraints. The unconstrained problem which minimises the overall training error using a cross entropy loss function is defined as:

8 $$\varepsilon = \frac{1}{n}\sum\nolimits_{i = 1}^{n} {y_{i} } \times \log \left( {\hat{y}_{i} } \right) + \left( {1 - y_{i} } \right) \times \log \left( {1 - \hat{y}_{i} } \right)$$

9 $$\begin{aligned} & {\mathcal{L}} = \varepsilon + \alpha_{1} p_{1} + \alpha_{2} p_{2} \\ & s.t. 0 \le m_{lj} \le 1 \\ \end{aligned}$$

where ${\alpha }_{1}$ and ${\alpha }_{2}$ denotes the strength of the penalties.

To ensure that relational preservation is maintained, the loss $\varepsilon$ can be penalised if the widths of gaussian membership functions (in the same UOD) significantly differ from each other. Using Eq. (6) to quantify the dissimilarity, if ${\sigma }_{lj}$ values are very different, the function will incur a higher penalty and if values ${\sigma }_{lj}$ are similar to each other the penalty will be smaller. Similarly for distinguishability, Eq. (7) can be used to incur penalties if membership functions have similar centres. The strength of the penalties can be adjusted with ${\alpha }_{1}, {\alpha }_{2}$ as hyperparameters. The conditions for relational preservation and distinguishability have been implemented as soft constraints and the problem can still be solved as a standard unconstrained optimisation problem. For bounded centres, the condition requires the satisfaction of the inequality constraint. In optimisation, inequality constraints can be handled with KKT (Karush–Kuhn–Tucker) or projected gradient-descent methods. However, projections onto the feasible region are computationally expensive and KKT conditions only handle simpler problems. Other methods use IF-ELSE conditions and clipping if constraints are violated. This approach is not always suitable, as the training is not very stable and the solutions are not optimal. Another way to handle inequality constraints for complex problems is to approximate the hard inequality as soft constraints through barrier methods. The violation of the inequality constraint is prevented by penalising the objective function if values get too close the boundary, forcing optimal solutions in the feasible region.

10 $$\begin{aligned} & c_{1} = - \frac{1}{t}\mathop \sum \limits_{l = 1}^{M} \mathop \sum \limits_{j = 1}^{D} \log \left( {m_{lj} } \right) \\ & c_{2} = - \frac{1}{t}\mathop \sum \limits_{l = 1}^{M} \mathop \sum \limits_{j = 1}^{D} \log \left( {1 - m_{lj} } \right) \\ & arg\,min {\mathcal{L}} = \varepsilon + \alpha_{1} p_{1} + \alpha_{2} p_{2} + c_{1} + c_{2} \\ \end{aligned}$$

The equations ${c}_{1}, {c}_{2}$ Eq. (10) are used to create a log barrier around feasible region (between 0 and 1) for values of ${m}_{lj}$. Values too close to the barrier will incur higher losses due to ${c}_{1}$ and ${c}_{2}$ thereby forcing the solution to stay within the feasible region. The log barrier with $t>0$ is a smooth approximation of the constraints with better approximation as $t\to \infty$. However, for large t, the gradient of the log-barrier can vary rapidly near the boundary of the feasible region. This can cause numerical issues and instability during optimization. In practice, the problem is solved via the central path, which starts with small values of *t* to solve the problem, and uses the solution as the starting point for the next iteration with increased value of *t*. As such, by following this method, the constrained optimisation problem can be solved with gradient based methods. The initial values are also required to be strictly feasible. The constrained optimisation problem can be solved by the following algorithm:
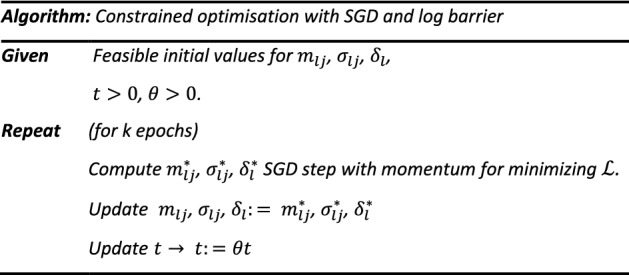


### Merging fuzzy sets for rule extraction

Depending on strength of the ${\alpha }_{1}$, ${\alpha }_{2}$ penalties during finetuning, there may be the case where one or two fuzzy sets are very similar to each other. Higher values for ${\alpha }_{2}$ will indeed result in fuzzy sets being different from each other as much as possible. However, this may also result in lower accuracies. If optimal values of ${\alpha }_{2}$ result in some fuzzy sets being very similar, we can merge these sets to further remove redundancy and simplify the rules. While this will not have a major effect on the prediction and inference, it may help improve the readability of the rules. In this research, to assess the similarity of the fine-tuned fuzzy sets, Jaccard similarity index is used as follows:

11 $$\begin{aligned} & S\left( {\mu_{Aj} ,\mu_{Bj} } \right) = \frac{{\mu_{Aj} \cap \mu_{Bj} }}{{\mu_{Aj} \cup \mu_{Bj} }} \\ & S\left( {\mu_{Aj} ,\mu_{Bj} } \right) = \frac{{\mathop \sum \nolimits_{x \in X} {\text{min}}\left\{ {\mu \left( x \right)_{Aj} ,\mu \left( x \right)_{Bj} } \right\}}}{{\mathop \sum \nolimits_{x \in X} {\text{max}}\left\{ {\mu \left( x \right)_{Aj} ,\mu \left( x \right)_{Bj} } \right\}}} \\ \end{aligned}$$

where ${\mu }_{Aj}$ and ${\mu }_{Bj}$ are the two fuzzy sets defined by gaussian membership functions for a particular feature $j$. The index shown in Eq. (11) is a non-analytical set theoretic based metric, therefore, similarity is computed by discretising the fuzzy sets. $X$ is the universe of discourse, ranged between 0 and 1 which is used to calculate the membership degrees defining the fuzzy sets. Fuzzy sets $A$ or $B$ which have a high degree of similarity close to 1, can be replaced by the either set. Alternatively, $A$ or $B$ can be merged to produce a new fuzzy set $C$. This can simply be done by taking $A \cup B$ as the support (width) of the new fuzzy set and by averaging the centres of $A$ and $B$ to obtain a new centre for $C$. This is only suitable for very similar sets, as merging distinct fuzzy sets may result in lower accuracy and non-optimal rules. After merging, rules can be extracted by using the gaussian centres $m$ as the rule antecedents and $\delta$ to determine which rule(s) are activated for a given class.

## Supplementary Information

Supplementary Information 1.

## Data Availability

The Bipolar dataset is available on the GEO database and can be accessed via https://www.ncbi.nlm.nih.gov/geo/query/acc.cgi?acc=GSE124326. The liver cancer (HCC) dataset is publicly available from https://www.ncbi.nlm.nih.gov/geo/query/acc.cgi?acc=GSE57957. However, the LYRIKS dataset is not publicly available due to participant consent statement but could be available from the corresponding author upon reasonable request and with permission of NTU and IMH, Singapore, considering a data sharing agreement procedure.

## Code availability

Code available upon request.
