# Supplementary material for: Constrained neuro fuzzy inference methodology for explainable personalised modelling with applications on gene expression data
Source: Sci Rep. 2023 Jan 9;13:456. doi: 10.1038/s41598-022-27132-8 (PMC9829920; doi:10.1038/s41598-022-27132-8)
Supplement: Supplementary file 1 — Supplementary Information 1. [file 41598_2022_27132_MOESM1_ESM.docx]

Constrained Neuro Fuzzy Inference Methodology for Explainable Personalised Modelling with Applications on Gene Expression Data

Balkaran Singh^1*^, Maryam Doborjeh^1*^, Zohreh Doborjeh^2,10^, Sugam Budhraja^1^, Samuel Tan^3^, Alexander Sumich^4^, Wilson Goh^3, 8,9^, Jimmy Lee^3,5^, Edmund Lai^1^, Nikola Kasabov^1,6,7^

^1^Knowledge Engineering and Discovery Research Innovation (KEDRI), School of Engineering Computer and Mathematical Sciences, Auckland University of Technology, New Zealand.

^2^School of Population Health, The University of Auckland, New Zealand.

^3^Lee Kong Chian School of Medicine, Nanyang Technological University (NTU), Singapore.

^4^Department of Psychology, Nottingham Trent University, Nottingham, UK.

^5^Institute for Mental Health, Singapore.

^6^Intelligent Systems Research Center, Ulster University, Derry, UK.

^7^Institute for Information and Communication Technologies, Bulgarian Academy of Sciences, Sofia, Bulgaria.

^8^Center for Biomedical Informatics, Nanyang Technological University (NTU), Singapore.

^9^School of Biological Sciences, Nanyang Technological University (NTU), Singapore.

^10^School of Psychology, The University Waikato, Hamilton, New Zealand.

*Contact person: [balkaran.singh@aut.ac.nz](mailto:balkaran.singh@aut.ac.nz) and [mgholami@aut.ac.nz](mailto:mgholami@aut.ac.nz)

# Supplementary Materials

Supplementary Figure S1


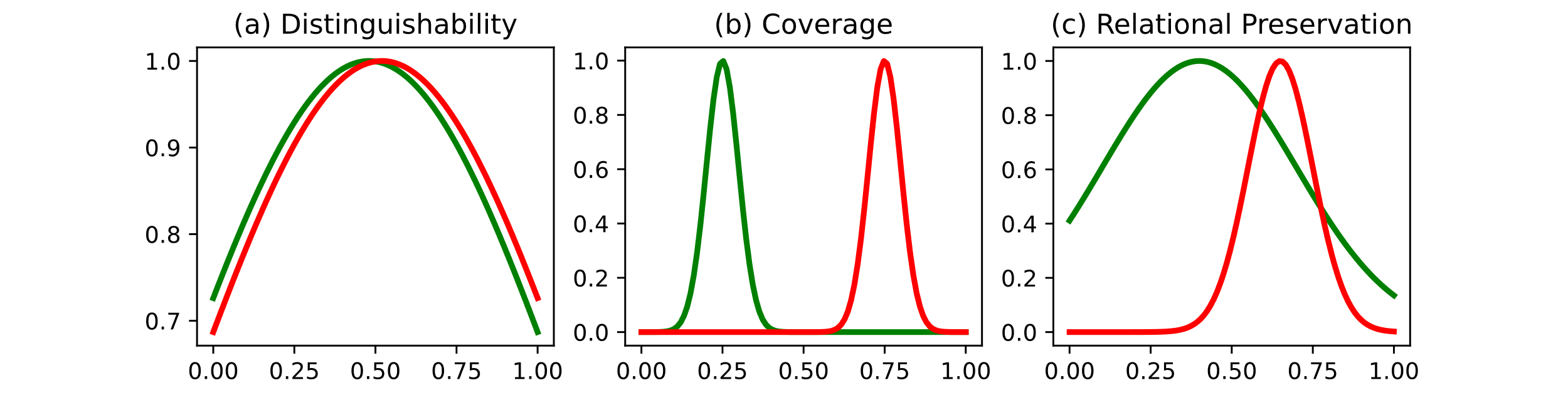


Supplementary Figure S1: Examples of violations in distinguishability, coverage, relational preservation conditions for interpretability. (a) since the two membership functions (shown in green and red) are very similar to each other, they do not refer to well distinguished concepts. (b) The two membership functions are completely disjoint and do not assign memberships to all elements of the universe of discourse (UOD). (c) The tails of a membership function are fully contained in another membership function.

Supplementary Figure S2


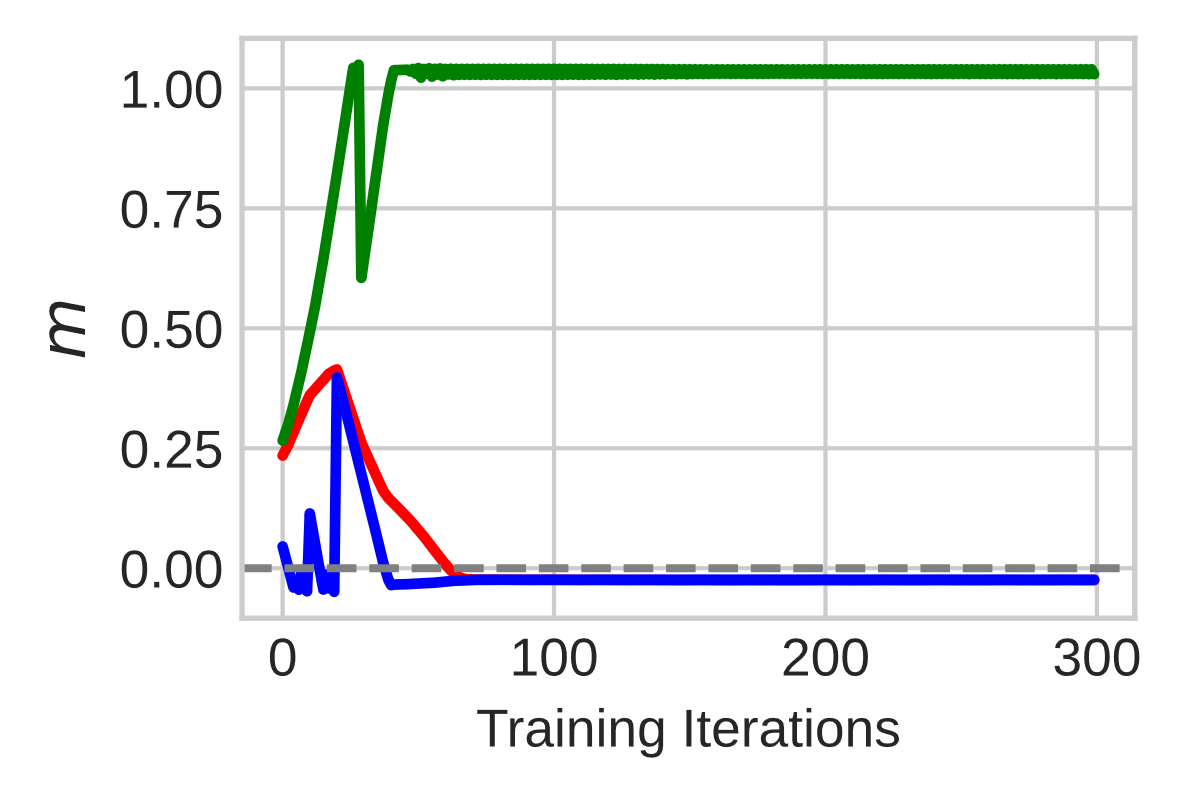

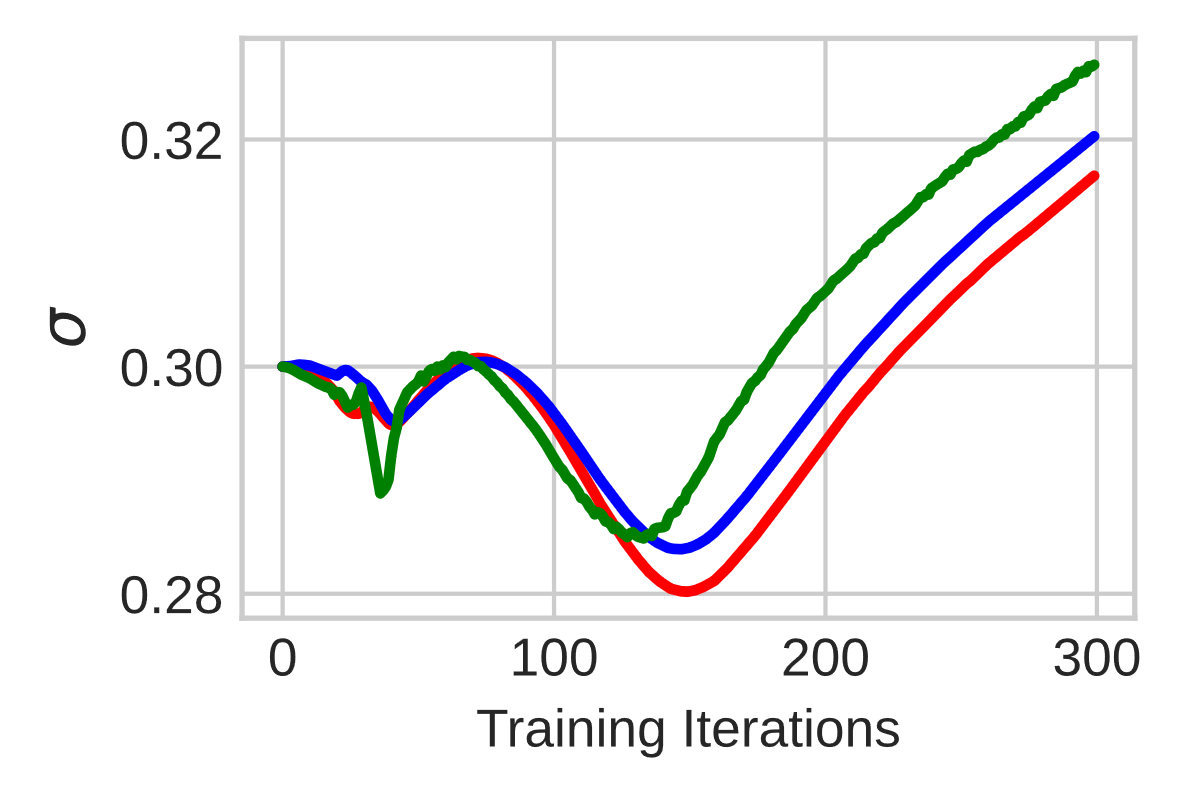

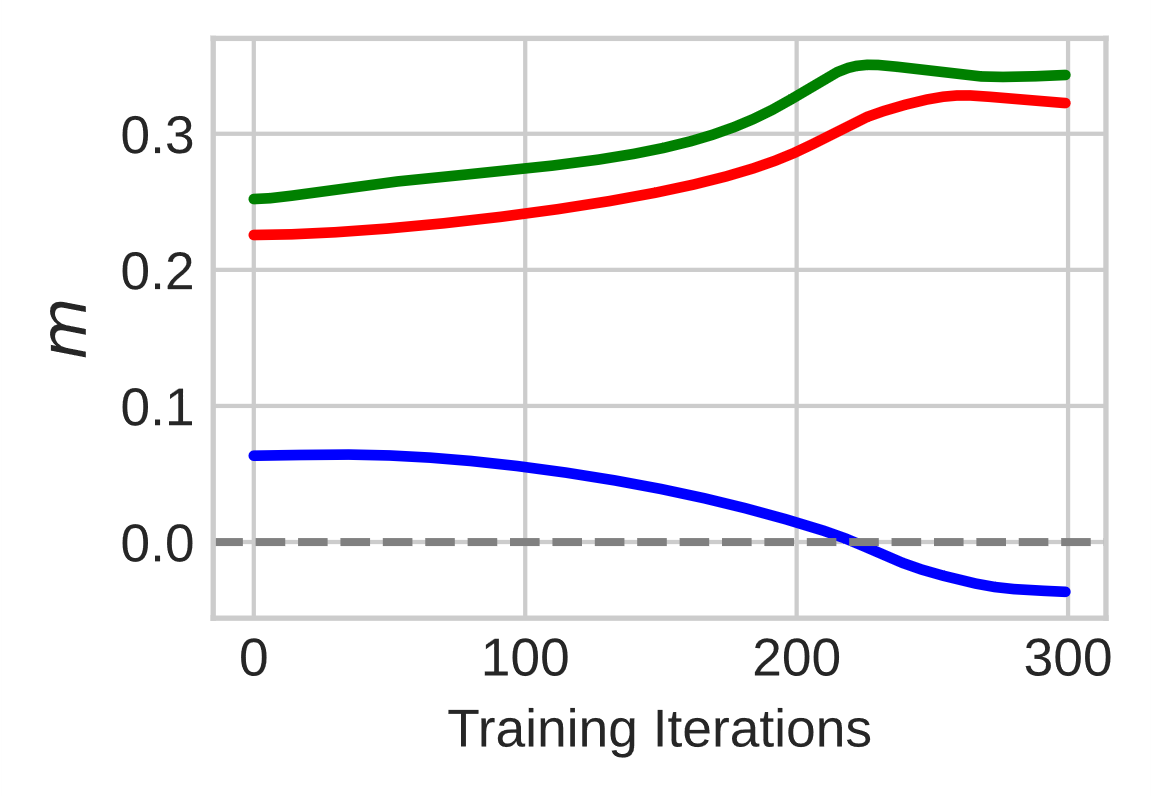

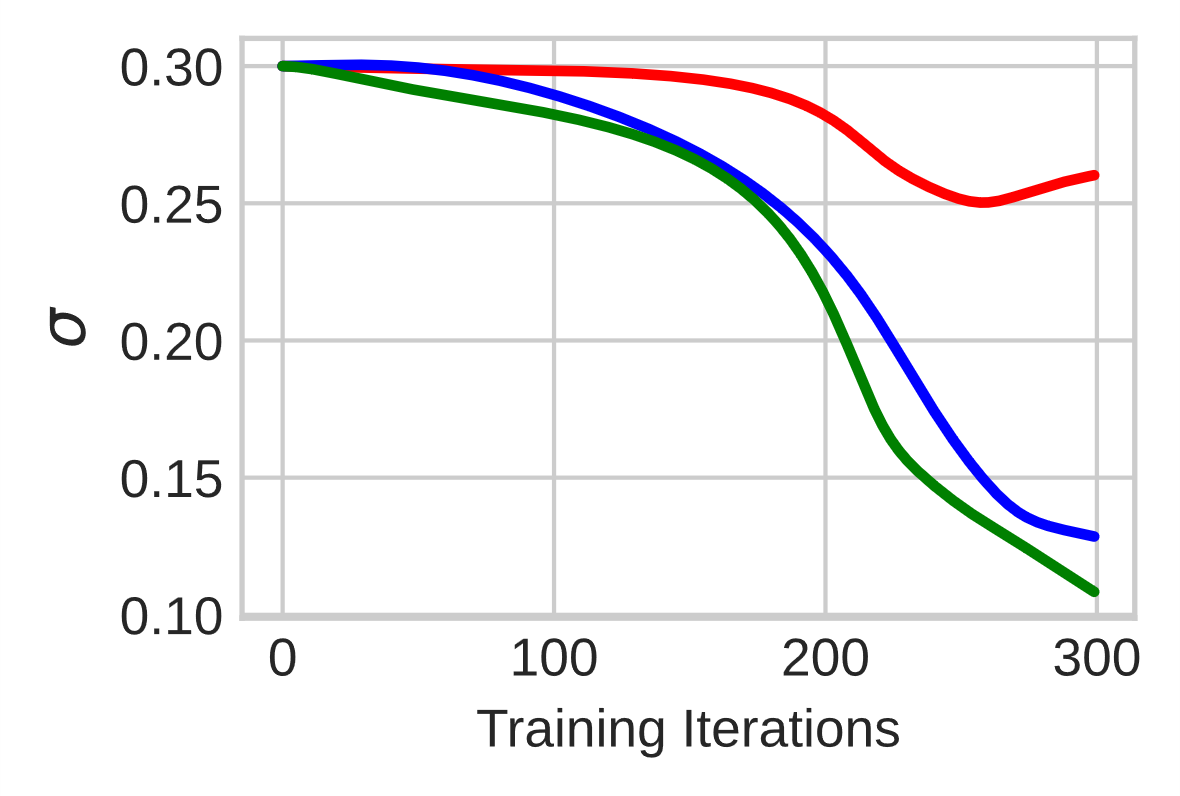

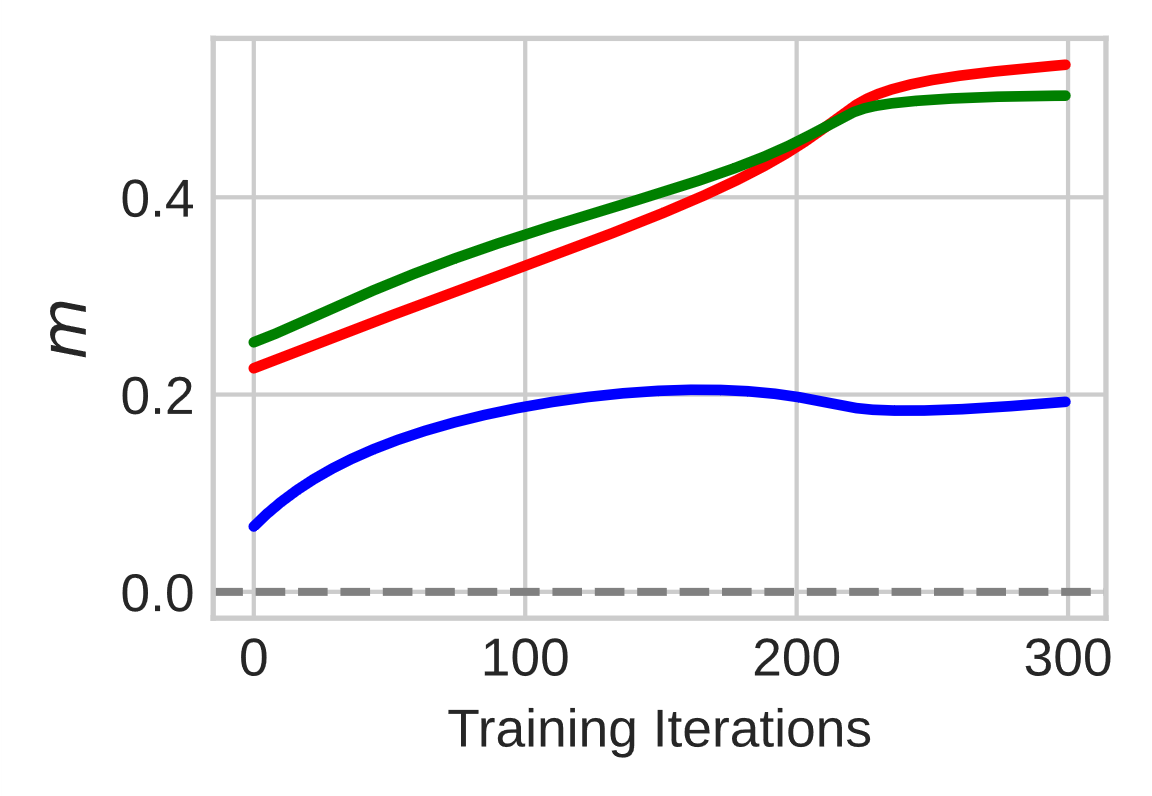

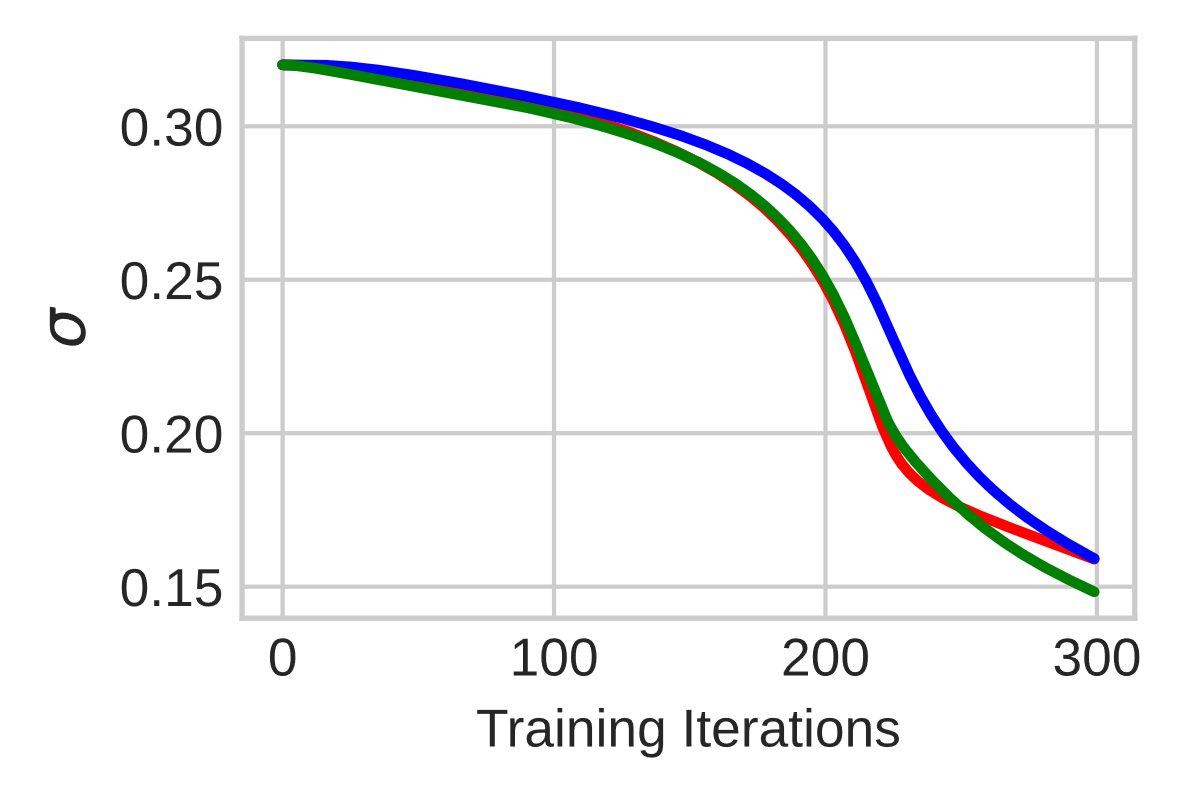


c) Non-optimal constraints

b) Optimal constraints

1. Unconstrained

*Supplementary Figure S2: The updates for the centre (m) and the width (*$\sigma$*) show that the m of the membership function in blue started as a positive number but became negative over the course of the optimisation process. Similarly, the* $\sigma$ *were initially similar but eventually became different leading to violation of interpretability conditions. In (b) the log barrier is preventing the m in blue from violating the range of the UOD (going below 0), while the penalties are forcing m to be distinct and* $\sigma$ *to be similar. In (c) the constraint strength is increased and this forcing m to be as distinct as possible and eventually plateau along the bounds of the log barrier.*

Supplementary Figure S3


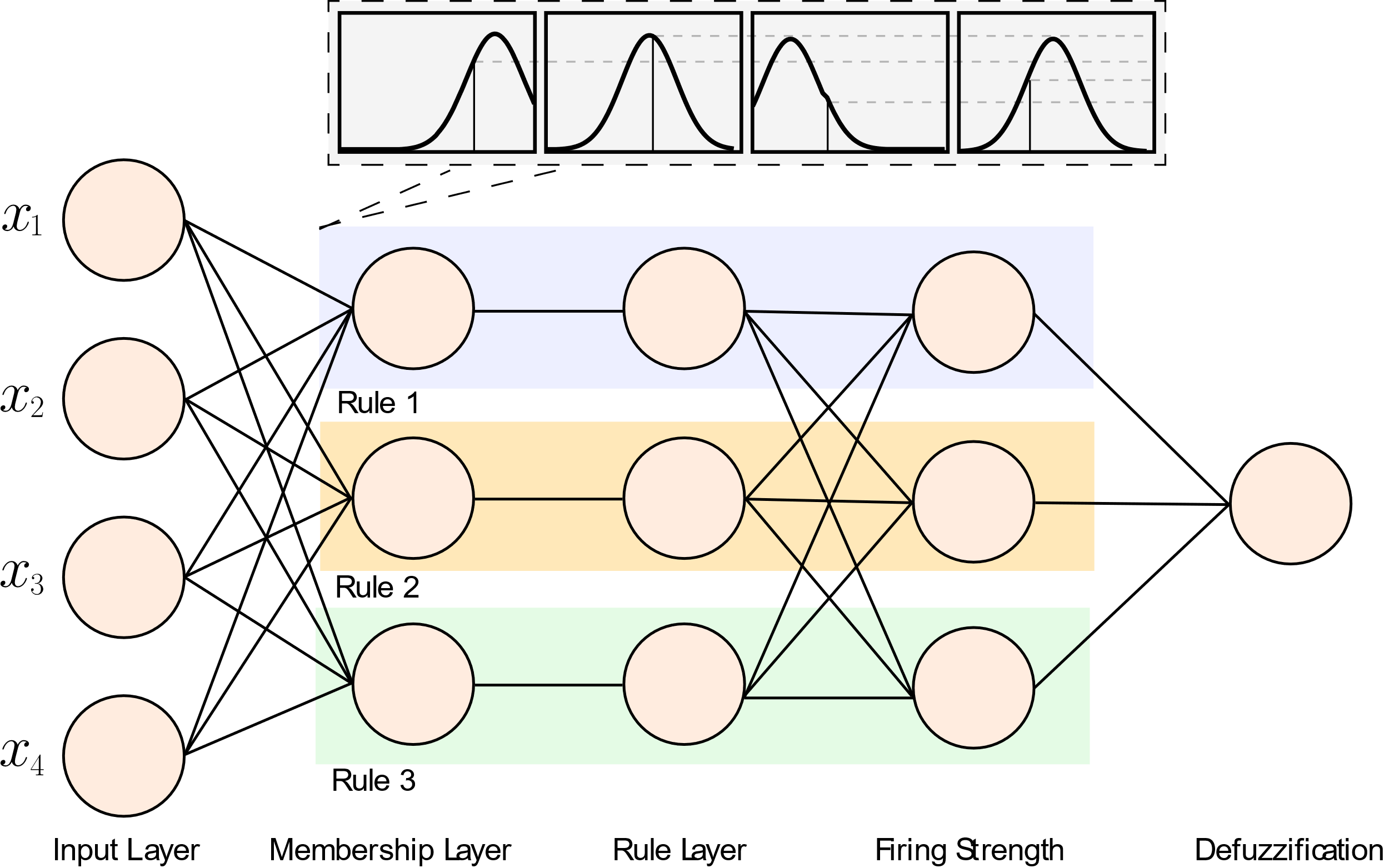


Supplementary Figure S3: An example of a generic NFIS with four input features $(x_{1}-x_{4})$ and three rules.

Supplementary Figure S4


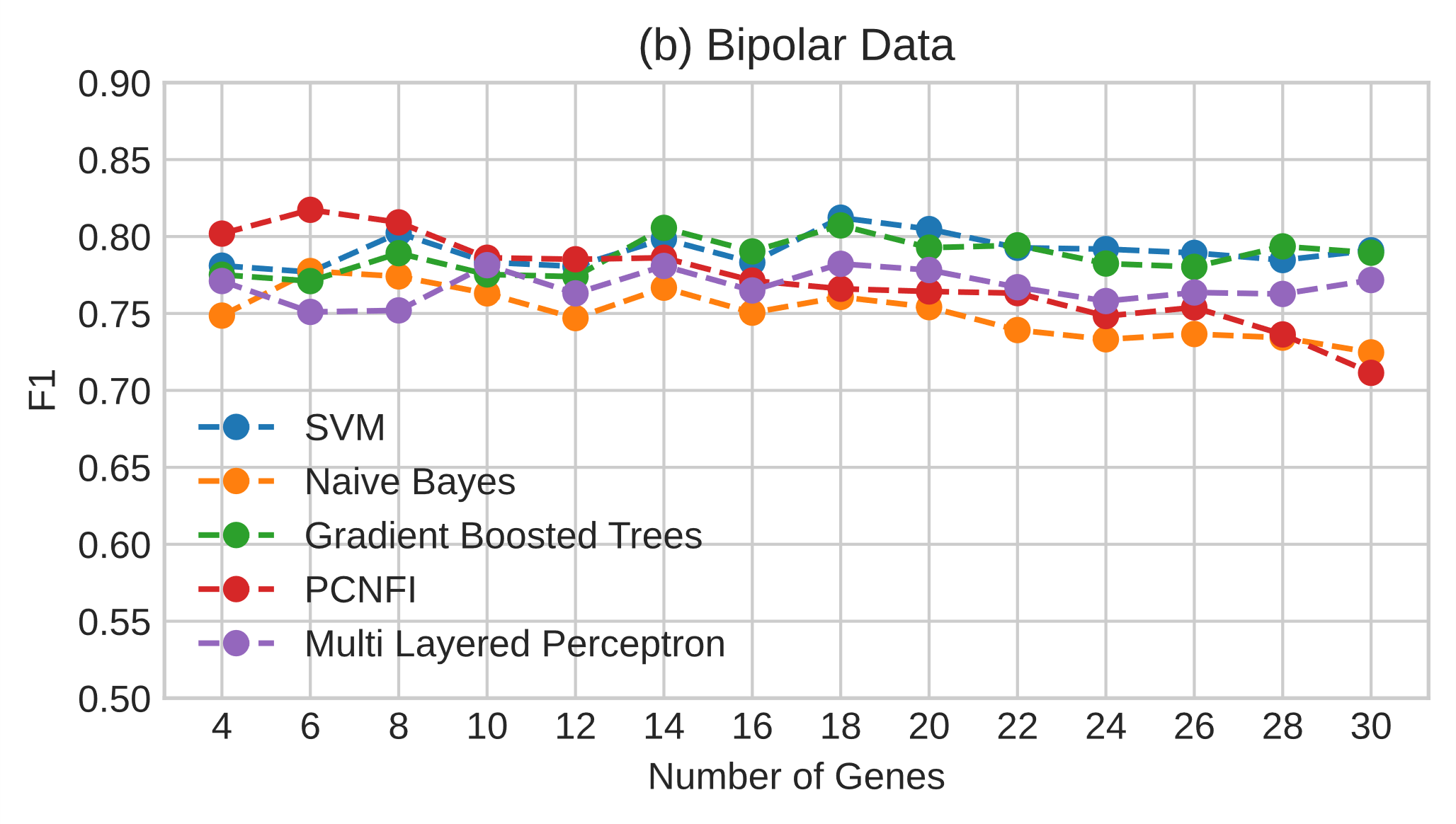

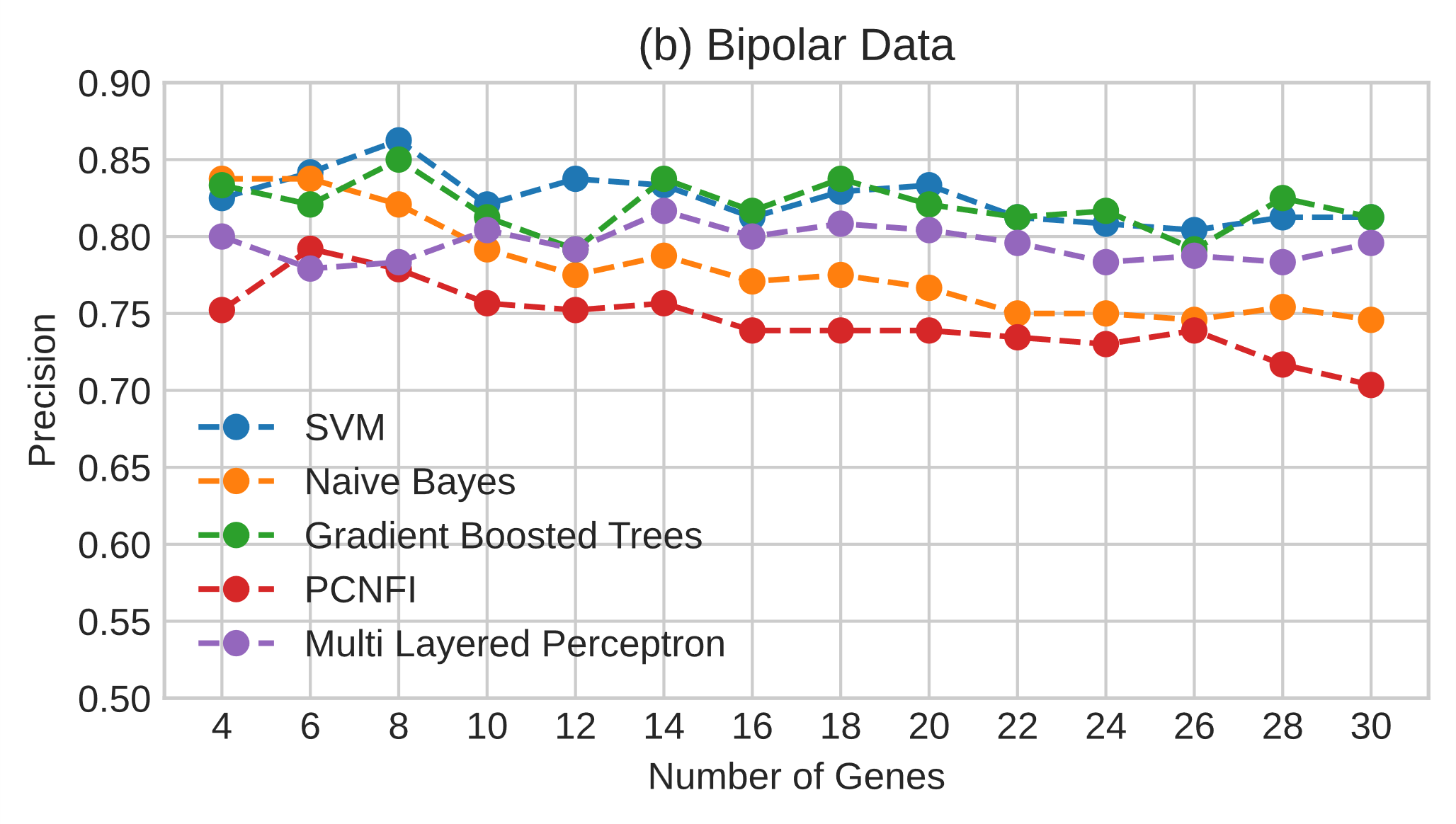

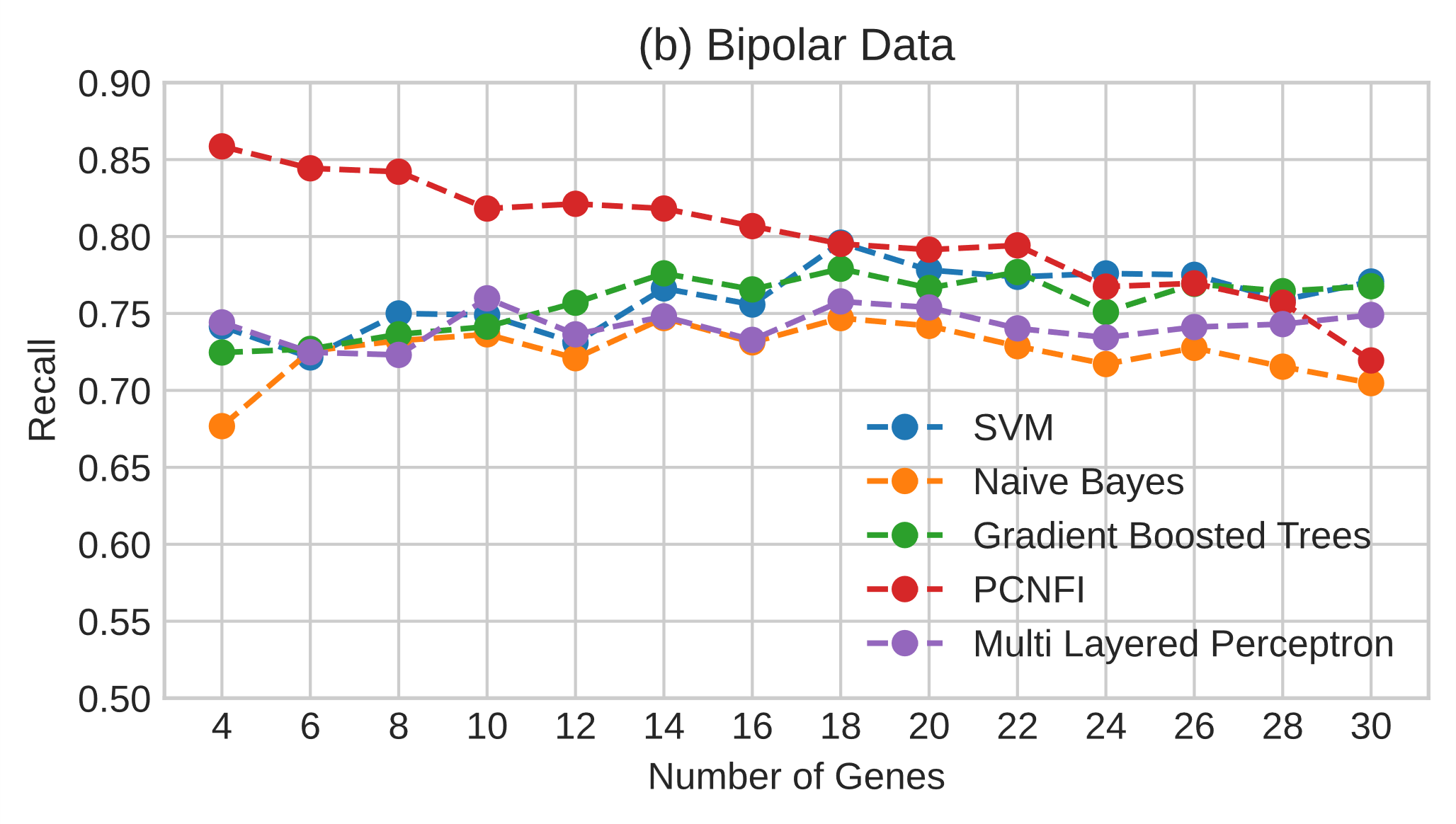

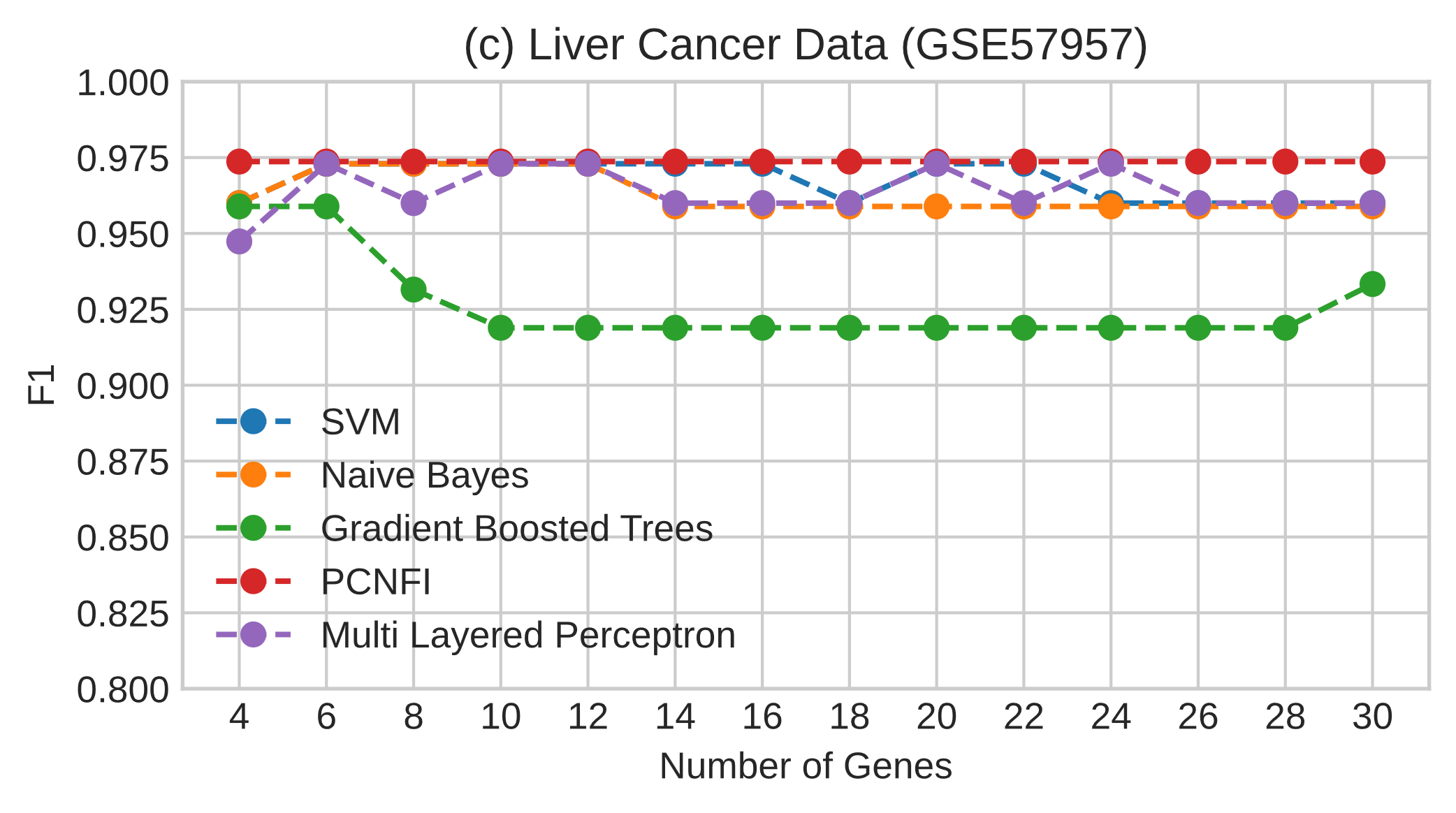

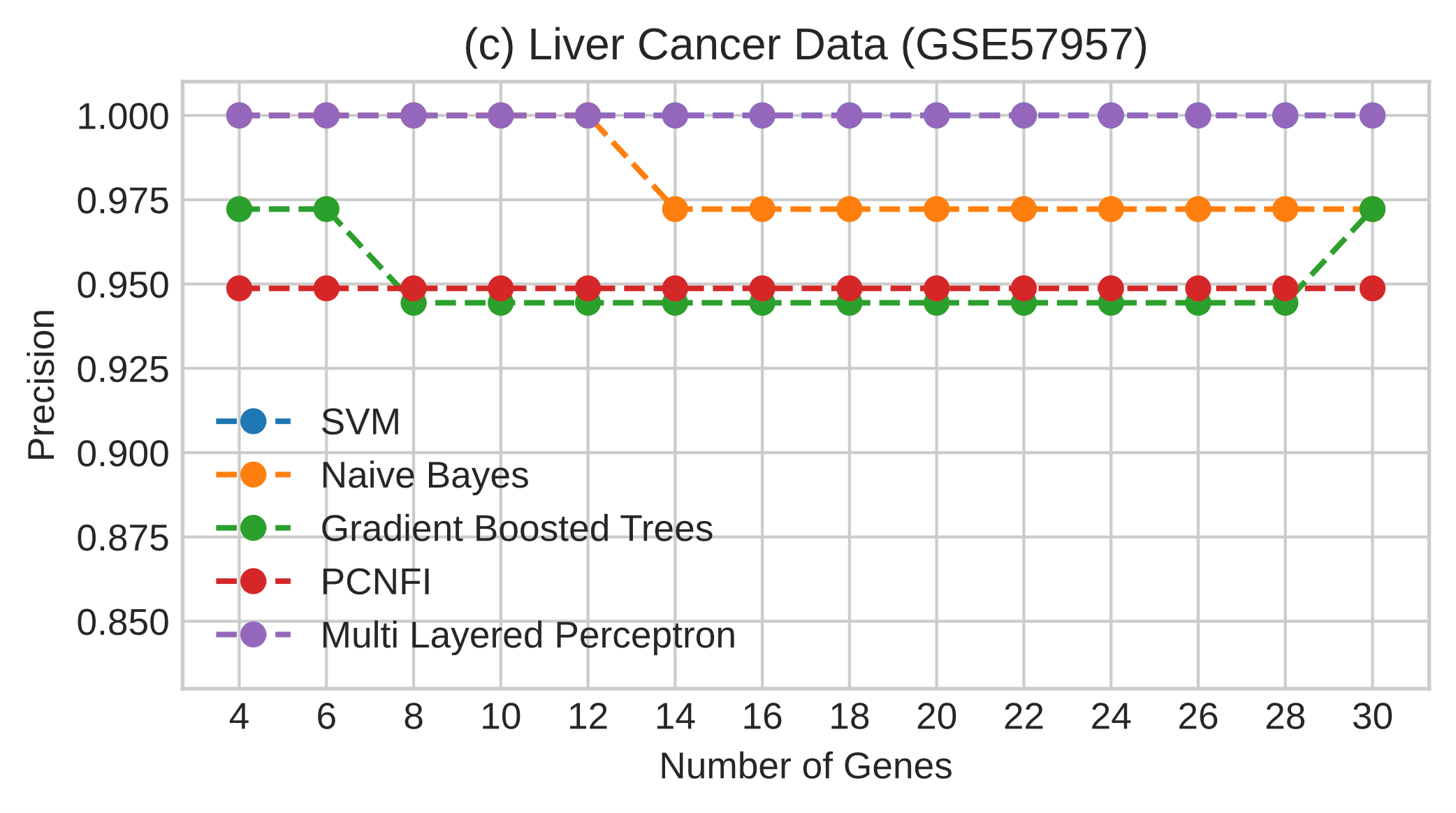

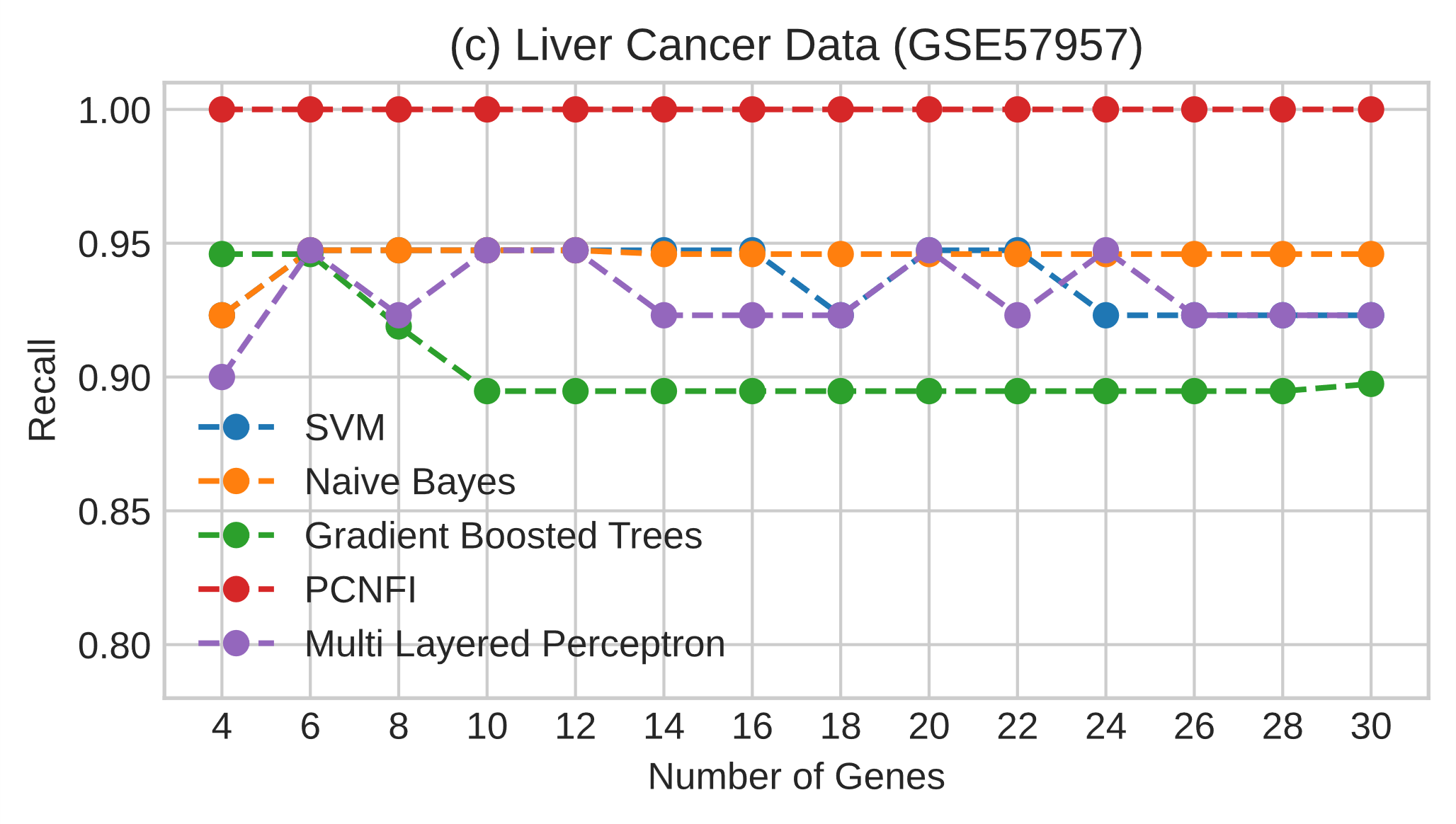

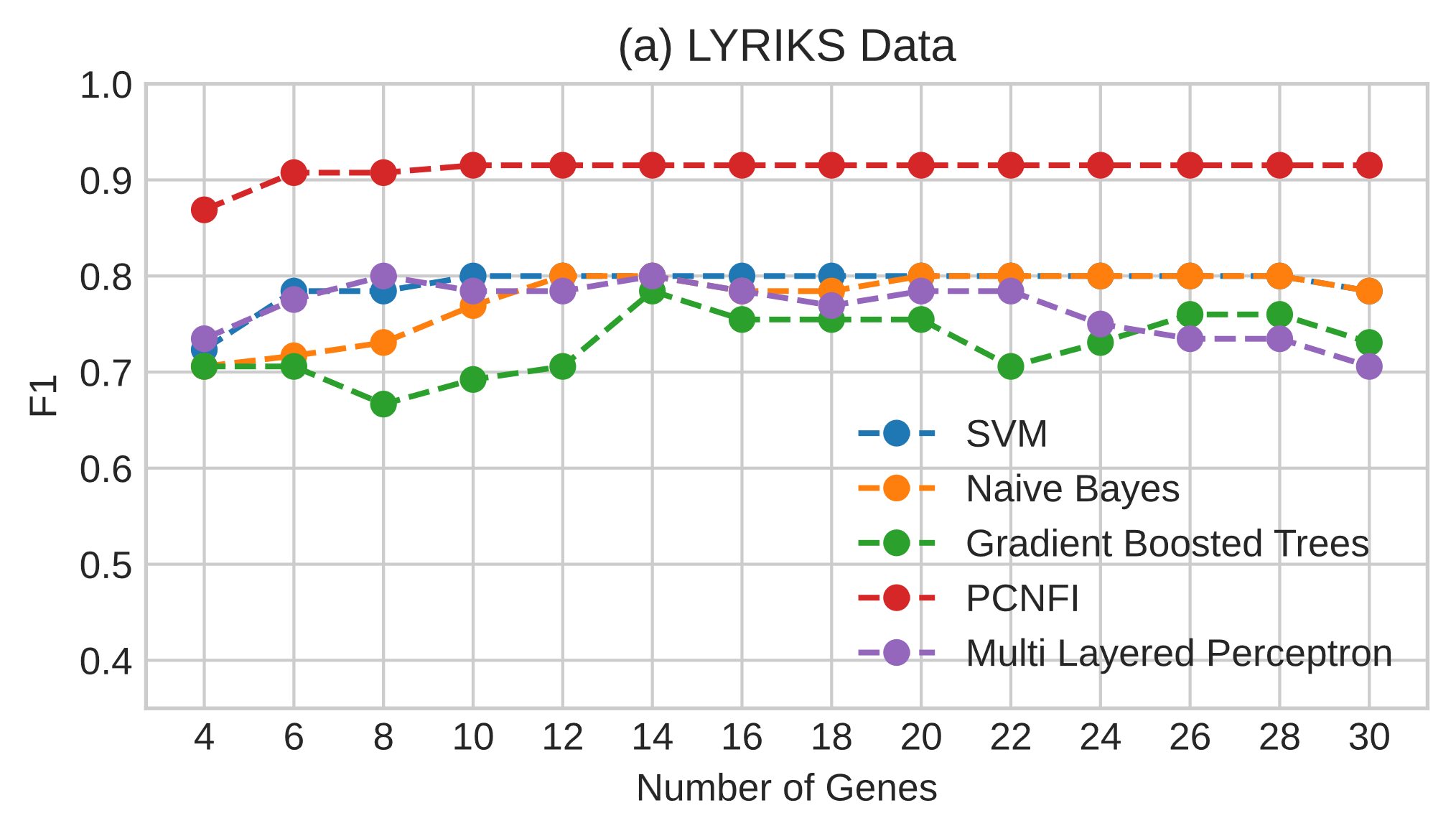

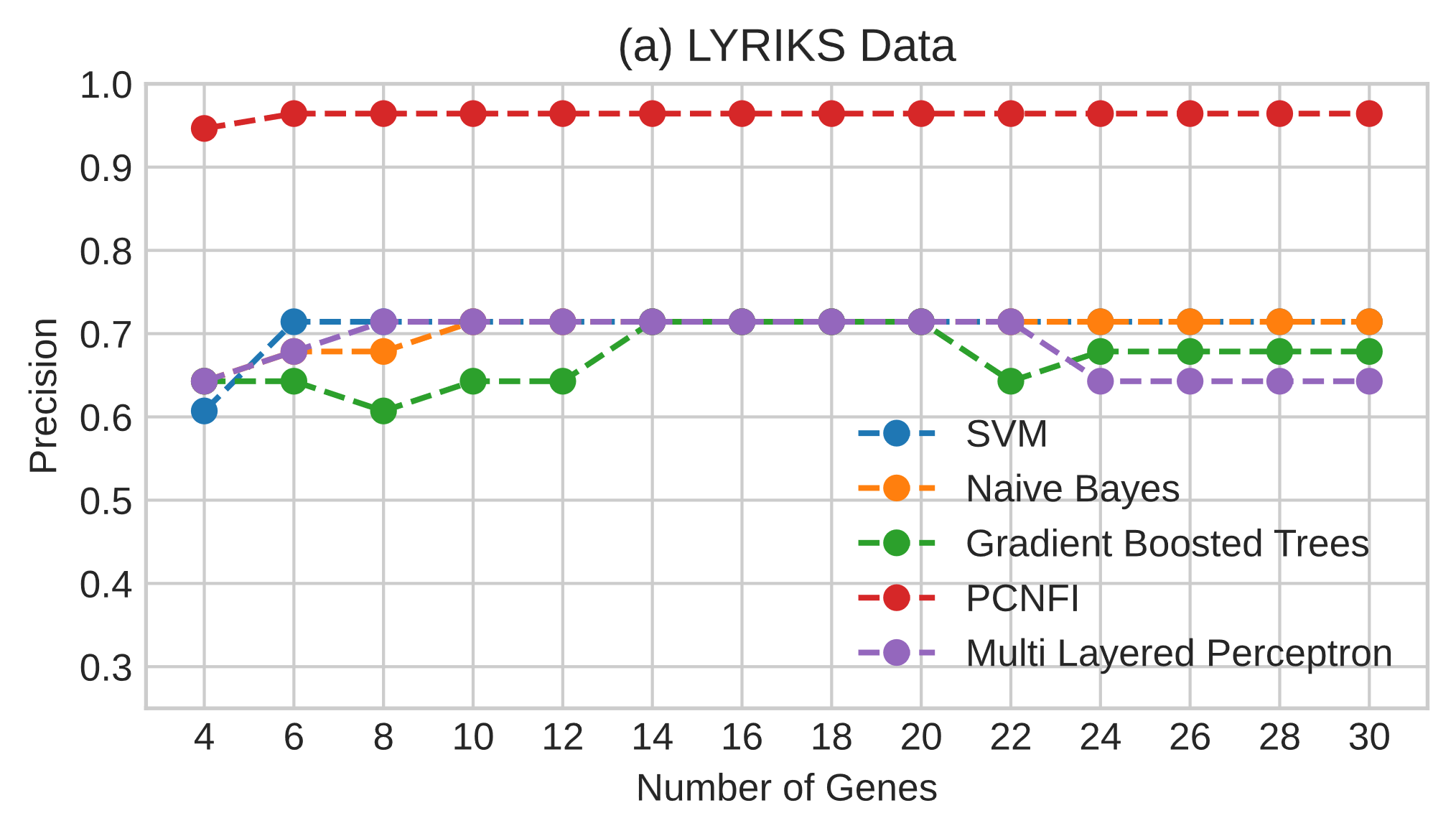

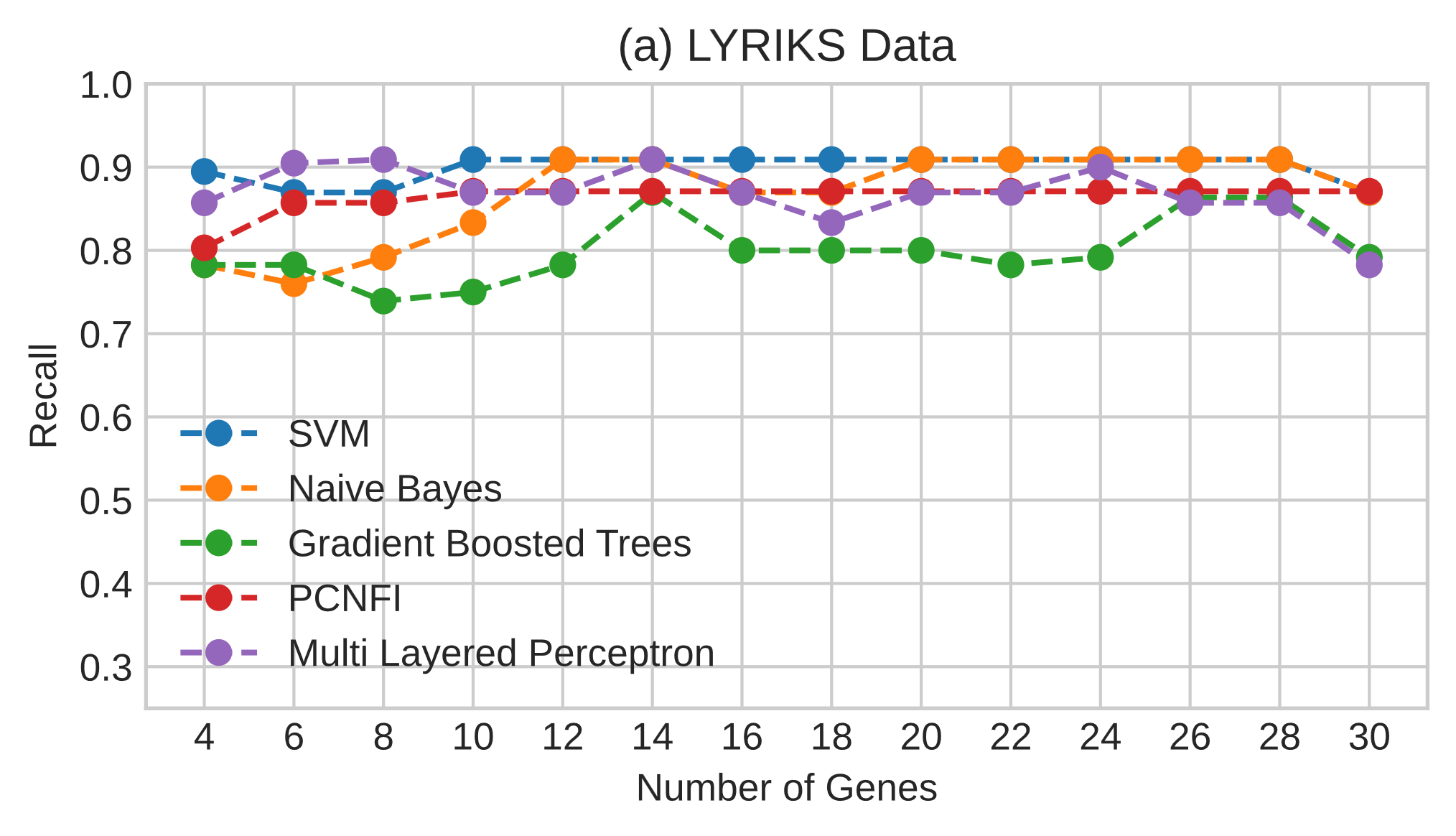


Supplementary Figure S4: F1 Score, Precision and Recall for (a) LYRIKS data (b) Bipolar data (c) Cancer data

Supplementary Table S1

|  | N | 4 | 6 | 8 | 10 | 12 | 14 | 16 | 18 | 20 | 22 | 24 | 26 | 28 | 30 |
| --- | --- | --- | --- | --- | --- | --- | --- | --- | --- | --- | --- | --- | --- | --- | --- |
| SVM | Acc | 0.85 | 0.87 | 0.87 | 0.88 | 0.88 | 0.88 | 0.88 | 0.88 | 0.88 | 0.88 | 0.88 | 0.88 | 0.88 | 0.87 |
|  | F1 | 0.72 | 0.78 | 0.78 | 0.8 | 0.8 | 0.8 | 0.8 | 0.8 | 0.8 | 0.8 | 0.8 | 0.8 | 0.8 | 0.78 |
|  | Pre | 0.61 | 0.71 | 0.71 | 0.71 | 0.71 | 0.71 | 0.71 | 0.71 | 0.71 | 0.71 | 0.71 | 0.71 | 0.71 | 0.71 |
|  | Rec | 0.89 | 0.87 | 0.87 | 0.91 | 0.91 | 0.91 | 0.91 | 0.91 | 0.91 | 0.91 | 0.91 | 0.91 | 0.91 | 0.87 |
| NB | Acc | 0.82 | 0.82 | 0.83 | 0.86 | 0.88 | 0.88 | 0.87 | 0.87 | 0.88 | 0.88 | 0.88 | 0.88 | 0.88 | 0.87 |
|  | F1 | 0.71 | 0.72 | 0.73 | 0.77 | 0.8 | 0.8 | 0.78 | 0.78 | 0.8 | 0.8 | 0.8 | 0.8 | 0.8 | 0.78 |
|  | Pre | 0.64 | 0.68 | 0.68 | 0.71 | 0.71 | 0.71 | 0.71 | 0.71 | 0.71 | 0.71 | 0.71 | 0.71 | 0.71 | 0.71 |
|  | Rec | 0.78 | 0.76 | 0.79 | 0.83 | 0.91 | 0.91 | 0.87 | 0.87 | 0.91 | 0.91 | 0.91 | 0.91 | 0.91 | 0.87 |
| GBT | Acc | 0.82 | 0.82 | 0.8 | 0.81 | 0.82 | 0.87 | 0.85 | 0.85 | 0.85 | 0.82 | 0.83 | 0.86 | 0.86 | 0.83 |
|  | F1 | 0.71 | 0.71 | 0.67 | 0.69 | 0.71 | 0.78 | 0.75 | 0.75 | 0.75 | 0.71 | 0.73 | 0.76 | 0.76 | 0.73 |
|  | Pre | 0.64 | 0.64 | 0.61 | 0.64 | 0.64 | 0.71 | 0.71 | 0.71 | 0.71 | 0.64 | 0.68 | 0.68 | 0.68 | 0.68 |
|  | Rec | 0.78 | 0.78 | 0.74 | 0.75 | 0.78 | 0.87 | 0.8 | 0.8 | 0.8 | 0.78 | 0.79 | 0.86 | 0.86 | 0.79 |
| PCNFI | Acc | 0.81 | 0.87 | 0.87 | 0.88 | 0.88 | 0.88 | 0.88 | 0.88 | 0.88 | 0.88 | 0.88 | 0.88 | 0.88 | 0.88 |
|  | F1 | 0.87 | 0.91 | 0.91 | 0.92 | 0.92 | 0.92 | 0.92 | 0.92 | 0.92 | 0.92 | 0.92 | 0.92 | 0.92 | 0.92 |
|  | Pre | 0.95 | 0.96 | 0.96 | 0.96 | 0.96 | 0.96 | 0.96 | 0.96 | 0.96 | 0.96 | 0.96 | 0.96 | 0.96 | 0.96 |
|  | Rec | 0.8 | 0.86 | 0.86 | 0.87 | 0.87 | 0.87 | 0.87 | 0.87 | 0.87 | 0.87 | 0.87 | 0.87 | 0.87 | 0.87 |
| MLP | Acc | 0.85 | 0.87 | 0.88 | 0.87 | 0.87 | 0.88 | 0.87 | 0.86 | 0.87 | 0.87 | 0.86 | 0.85 | 0.85 | 0.82 |
|  | F1 | 0.73 | 0.78 | 0.8 | 0.78 | 0.78 | 0.8 | 0.78 | 0.77 | 0.78 | 0.78 | 0.75 | 0.73 | 0.73 | 0.71 |
|  | Pre | 0.64 | 0.68 | 0.71 | 0.71 | 0.71 | 0.71 | 0.71 | 0.71 | 0.71 | 0.71 | 0.64 | 0.64 | 0.64 | 0.64 |
|  | Rec | 0.86 | 0.9 | 0.91 | 0.87 | 0.87 | 0.91 | 0.87 | 0.83 | 0.87 | 0.87 | 0.9 | 0.86 | 0.86 | 0.78 |

Supplementary Table S1: Performance comparison of PCNFI and other classifiers on the LYRIKS data

Supplementary Table S2

|  | N | 4 | 6 | 8 | 10 | 12 | 14 | 16 | 18 | 20 | 22 | 24 | 26 | 28 | 30 |
| --- | --- | --- | --- | --- | --- | --- | --- | --- | --- | --- | --- | --- | --- | --- | --- |
| SVM | Acc | 0.76 | 0.75 | 0.78 | 0.77 | 0.76 | 0.78 | 0.77 | 0.8 | 0.79 | 0.78 | 0.78 | 0.78 | 0.77 | 0.78 |
|  | F1 | 0.78 | 0.78 | 0.8 | 0.78 | 0.78 | 0.8 | 0.78 | 0.81 | 0.8 | 0.79 | 0.79 | 0.79 | 0.78 | 0.79 |
|  | Pre | 0.82 | 0.84 | 0.86 | 0.82 | 0.84 | 0.83 | 0.81 | 0.83 | 0.83 | 0.81 | 0.81 | 0.8 | 0.81 | 0.81 |
|  | Rec | 0.74 | 0.72 | 0.75 | 0.75 | 0.73 | 0.77 | 0.76 | 0.8 | 0.78 | 0.77 | 0.78 | 0.78 | 0.76 | 0.77 |
| NB | Acc | 0.71 | 0.75 | 0.75 | 0.75 | 0.73 | 0.75 | 0.74 | 0.75 | 0.74 | 0.73 | 0.72 | 0.73 | 0.72 | 0.71 |
|  | F1 | 0.75 | 0.78 | 0.77 | 0.76 | 0.75 | 0.77 | 0.75 | 0.76 | 0.75 | 0.74 | 0.73 | 0.74 | 0.73 | 0.72 |
|  | Pre | 0.84 | 0.84 | 0.82 | 0.79 | 0.78 | 0.79 | 0.77 | 0.78 | 0.77 | 0.75 | 0.75 | 0.75 | 0.75 | 0.75 |
|  | Rec | 0.68 | 0.73 | 0.73 | 0.74 | 0.72 | 0.75 | 0.73 | 0.75 | 0.74 | 0.73 | 0.72 | 0.73 | 0.72 | 0.7 |
| GBT | Acc | 0.75 | 0.75 | 0.77 | 0.76 | 0.76 | 0.79 | 0.78 | 0.79 | 0.78 | 0.78 | 0.77 | 0.77 | 0.78 | 0.78 |
|  | F1 | 0.78 | 0.77 | 0.79 | 0.78 | 0.77 | 0.81 | 0.79 | 0.81 | 0.79 | 0.79 | 0.78 | 0.78 | 0.79 | 0.79 |
|  | Pre | 0.83 | 0.82 | 0.85 | 0.81 | 0.79 | 0.84 | 0.82 | 0.84 | 0.82 | 0.81 | 0.82 | 0.79 | 0.82 | 0.81 |
|  | Rec | 0.72 | 0.73 | 0.74 | 0.74 | 0.76 | 0.78 | 0.77 | 0.78 | 0.77 | 0.78 | 0.75 | 0.77 | 0.76 | 0.77 |
| PCNFI | Acc | 0.82 | 0.83 | 0.82 | 0.8 | 0.8 | 0.8 | 0.79 | 0.78 | 0.78 | 0.78 | 0.76 | 0.77 | 0.75 | 0.72 |
|  | F1 | 0.8 | 0.82 | 0.81 | 0.79 | 0.79 | 0.79 | 0.77 | 0.77 | 0.76 | 0.76 | 0.75 | 0.75 | 0.74 | 0.71 |
|  | Pre | 0.75 | 0.79 | 0.78 | 0.76 | 0.75 | 0.76 | 0.74 | 0.74 | 0.74 | 0.73 | 0.73 | 0.74 | 0.72 | 0.7 |
|  | Rec | 0.86 | 0.84 | 0.84 | 0.82 | 0.82 | 0.82 | 0.81 | 0.8 | 0.79 | 0.79 | 0.77 | 0.77 | 0.76 | 0.72 |
| MLP | Acc | 0.76 | 0.73 | 0.73 | 0.77 | 0.75 | 0.76 | 0.75 | 0.77 | 0.76 | 0.75 | 0.74 | 0.75 | 0.75 | 0.76 |
|  | F1 | 0.77 | 0.75 | 0.75 | 0.78 | 0.76 | 0.78 | 0.76 | 0.78 | 0.78 | 0.77 | 0.76 | 0.76 | 0.76 | 0.77 |
|  | Pre | 0.8 | 0.78 | 0.78 | 0.8 | 0.79 | 0.82 | 0.8 | 0.81 | 0.8 | 0.8 | 0.78 | 0.79 | 0.78 | 0.8 |
|  | Rec | 0.74 | 0.72 | 0.72 | 0.76 | 0.74 | 0.75 | 0.73 | 0.76 | 0.75 | 0.74 | 0.73 | 0.74 | 0.74 | 0.75 |

Supplementary Table S2: Performance comparison of PCNFI and other classifiers on the Bipolar data

Supplementary Table S3

|  | N | 4 | 6 | 8 | 10 | 12 | 14 | 16 | 18 | 20 | 22 | 24 | 26 | 28 | 30 |
| --- | --- | --- | --- | --- | --- | --- | --- | --- | --- | --- | --- | --- | --- | --- | --- |
| SVM | Acc | 0.96 | 0.97 | 0.97 | 0.97 | 0.97 | 0.97 | 0.97 | 0.96 | 0.97 | 0.97 | 0.96 | 0.96 | 0.96 | 0.96 |
|  | F1 | 0.96 | 0.97 | 0.97 | 0.97 | 0.97 | 0.97 | 0.97 | 0.96 | 0.97 | 0.97 | 0.96 | 0.96 | 0.96 | 0.96 |
|  | Pre | 1 | 1 | 1 | 1 | 1 | 1 | 1 | 1 | 1 | 1 | 1 | 1 | 1 | 1 |
|  | Rec | 0.92 | 0.95 | 0.95 | 0.95 | 0.95 | 0.95 | 0.95 | 0.92 | 0.95 | 0.95 | 0.92 | 0.92 | 0.92 | 0.92 |
| NB | Acc | 0.96 | 0.97 | 0.97 | 0.97 | 0.97 | 0.96 | 0.96 | 0.96 | 0.96 | 0.96 | 0.96 | 0.96 | 0.96 | 0.96 |
|  | F1 | 0.96 | 0.97 | 0.97 | 0.97 | 0.97 | 0.96 | 0.96 | 0.96 | 0.96 | 0.96 | 0.96 | 0.96 | 0.96 | 0.96 |
|  | Pre | 1 | 1 | 1 | 1 | 1 | 0.97 | 0.97 | 0.97 | 0.97 | 0.97 | 0.97 | 0.97 | 0.97 | 0.97 |
|  | Rec | 0.92 | 0.95 | 0.95 | 0.95 | 0.95 | 0.95 | 0.95 | 0.95 | 0.95 | 0.95 | 0.95 | 0.95 | 0.95 | 0.95 |
| GBT | Acc | 0.96 | 0.96 | 0.93 | 0.92 | 0.92 | 0.92 | 0.92 | 0.92 | 0.92 | 0.92 | 0.92 | 0.92 | 0.92 | 0.93 |
|  | F1 | 0.96 | 0.96 | 0.93 | 0.92 | 0.92 | 0.92 | 0.92 | 0.92 | 0.92 | 0.92 | 0.92 | 0.92 | 0.92 | 0.93 |
|  | Pre | 0.97 | 0.97 | 0.94 | 0.94 | 0.94 | 0.94 | 0.94 | 0.94 | 0.94 | 0.94 | 0.94 | 0.94 | 0.94 | 0.97 |
|  | Rec | 0.95 | 0.95 | 0.92 | 0.89 | 0.89 | 0.89 | 0.89 | 0.89 | 0.89 | 0.89 | 0.89 | 0.89 | 0.89 | 0.9 |
| PCNFI | Acc | 0.97 | 0.97 | 0.97 | 0.97 | 0.97 | 0.97 | 0.97 | 0.97 | 0.97 | 0.97 | 0.97 | 0.97 | 0.97 | 0.97 |
|  | F1 | 0.97 | 0.97 | 0.97 | 0.97 | 0.97 | 0.97 | 0.97 | 0.97 | 0.97 | 0.97 | 0.97 | 0.97 | 0.97 | 0.97 |
|  | Pre | 0.95 | 0.95 | 0.95 | 0.95 | 0.95 | 0.95 | 0.95 | 0.95 | 0.95 | 0.95 | 0.95 | 0.95 | 0.95 | 0.95 |
|  | Rec | 1.0 | 1.0 | 1.0 | 1.0 | 1.0 | 1.0 | 1.0 | 1.0 | 1.0 | 1.0 | 1.0 | 1.0 | 1.0 | 1.0 |
| MLP | Acc | 0.95 | 0.97 | 0.96 | 0.97 | 0.97 | 0.96 | 0.96 | 0.96 | 0.97 | 0.96 | 0.97 | 0.96 | 0.96 | 0.96 |
|  | F1 | 0.95 | 0.97 | 0.96 | 0.97 | 0.97 | 0.96 | 0.96 | 0.96 | 0.97 | 0.96 | 0.97 | 0.96 | 0.96 | 0.96 |
|  | Pre | 1 | 1 | 1 | 1 | 1 | 1 | 1 | 1 | 1 | 1 | 1 | 1 | 1 | 1 |
|  | Rec | 0.9 | 0.95 | 0.92 | 0.95 | 0.95 | 0.92 | 0.92 | 0.92 | 0.95 | 0.92 | 0.95 | 0.92 | 0.92 | 0.92 |

Supplementary Table S3: Performance comparison of PCNFI and other classifiers on the Liver cancer data
